# Applying single cell multi-omic analyses to understand treatment resistance in pediatric high grade glioma

**DOI:** 10.3389/fphar.2023.1002296

**Published:** 2023-05-03

**Authors:** Rebecca L. Murdaugh, Jamie N. Anastas

**Affiliations:** ^1^ Department of Neurosurgery, Baylor College of Medicine, Houston, TX, United States; ^2^ Program in Cell and Gene Therapy, Baylor College of Medicine, Houston, TX, United States; ^3^ Department of Molecular and Cellular Biology, Baylor College of Medicine, Houston, TX, United States

**Keywords:** tumor hetereogeneity, clonal expansion, multi-omic, single cell profiling, drug resistance, pediatric high grade glioma, diffuse midline glioma

## Abstract

Despite improvements in cancer patient outcomes seen in the past decade, tumor resistance to therapy remains a major impediment to achieving durable clinical responses. Intratumoral heterogeneity related to genetic, epigenetic, transcriptomic, proteomic, and metabolic differences between individual cancer cells has emerged as a driver of therapeutic resistance. This cell to cell heterogeneity can be assessed using single cell profiling technologies that enable the identification of tumor cell clones that exhibit similar defining features like specific mutations or patterns of DNA methylation. Single cell profiling of tumors before and after treatment can generate new insights into the cancer cell characteristics that confer therapeutic resistance by identifying intrinsically resistant sub-populations that survive treatment and by describing new cellular features that emerge post-treatment due to tumor cell evolution. Integrative, single cell analytical approaches have already proven advantageous in studies characterizing treatment-resistant clones in cancers where pre- and post-treatment patient samples are readily available, such as leukemia. In contrast, little is known about other cancer subtypes like pediatric high grade glioma, a class of heterogeneous, malignant brain tumors in children that rapidly develop resistance to multiple therapeutic modalities, including chemotherapy, immunotherapy, and radiation. Leveraging single cell multi-omic technologies to analyze naïve and therapy-resistant glioma may lead to the discovery of novel strategies to overcome treatment resistance in brain tumors with dismal clinical outcomes. In this review, we explore the potential for single cell multi-omic analyses to reveal mechanisms of glioma resistance to therapy and discuss opportunities to apply these approaches to improve long-term therapeutic response in pediatric high grade glioma and other brain tumors with limited treatment options.

## Introduction

High grade glioma (HGG) are aggressive and highly malignant tumors of the central nervous system (Wen and Kesari, 2008; Cohen, 2022) and little progress has been made in improving clinical outcomes for these patients in the past 40 years. Survival rates are low in both pediatric and adult HGG, and certain subtypes of pediatric HGG (pHGG), like diffuse midline glioma (DMG), are almost universally lethal (Jones et al., 2017; Ostrom et al., 2019). Our poor understanding of which processes and pathways drive pHGG malignancy and response to therapy has impeded efforts to develop effective treatments for pHGG and other brain tumors. Pan-cancer analyses suggest the presence of up to 1,000-fold fewer mutations in pediatric tumors compared to adult tumors arising in the same tissue (ICGC PedBrain-Seq Project et al., 2018; Ma et al., 2018), and certain driver mutations, including genetic aberrations in epigenetic regulators like histone H3 point mutations (H3K27M and H3G34 R/V), are observed more frequently in pediatric glioma compared to adult brain tumors (Khuong-Quang et al., 2012; Schwartzentruber et al., 2012; Wu et al., 2012; Fontebasso et al., 2013; Wiestler et al., 2013; Mackay et al., 2017). The divergent genetic profile of pHGG tumors suggests that focused studies aimed at identifying mediators of therapeutic response specific to pediatric cohorts have the potential to inform personalized strategies to improve clinical outcomes.

The abysmal survival rate seen in adult HGG is often attributed to these tumors exhibiting stem cell-like phenotypes associated with poor response to radiation, temozolomide (TMZ) and other therapies (Bao et al., 2006; Liu et al., 2006; Chen et al., 2012; Alexander et al., 2020; Petralia et al., 2020; Syafruddin et al., 2021; Wang et al., 2021). Despite potential roles for rare tumor sub-populations in treatment resistance, most efforts to characterize glioma response to therapy rely on either limited tumor gene panels or bulk sequencing to compare tumor samples from different patients (intertumoral heterogeneity) rather than addressing cell to cell variation within individual tumors (intratumoral heterogeneity) (Allen et al., 2017; Mueller et al., 2019; Nejo et al., 2019; Petralia et al., 2020; Wang et al., 2021). Recent single cell analyses highlight the strikingly heterogeneous nature of glioma tumors and have enabled the profiling of glioma stem and progenitor like cells, the analysis of lineage diversity among tumor cells, including mesenchymal, oligodendroglial, and astrocytic sub-populations (Sottoriva et al., 2013; Darmanis et al., 2017; Filbin et al., 2018; Yuan et al., 2018; Neftel et al., 2019; Zhai et al., 2020; Guilhamon et al., 2021; Abdelfattah et al., 2022; Curry et al., 2023; Khan et al., 2023). It is also possible that the stem cell-like and therapy-resistant properties of HGG are conferred by a relatively small number of tumor cells, which cannot be easily detected or fully characterized by bulk sequencing (Bao et al., 2006; Liu et al., 2006; Chen et al., 2012; Alexander et al., 2020). Further single cell analyses highlight spatial differences in HGG cell phenotypes within different tumor regions (Sottoriva et al., 2013; Darmanis et al., 2017; Comba et al., 2021) and identify diverse tumor-associated immune cells suggesting that heterogeneity in the local tumor microenvironment may also play a key role in treatment resistance. Similarly applying single cell analyses to pediatric HGG before and after treatment has the potential to generate a more comprehensive view of the unique cellular composition of pediatric gliomas and their dynamic responses to therapy.

Because of technological limitations, most initial attempts at using single cell technologies to dissect glioma heterogeneity have profiled only one layer of biological regulation in isolation, such as cell to cell variation in transcription through single cell RNA sequencing (scRNAseq). Since cellular heterogeneity can occur at the level of the genome, epigenome, transcriptome, proteome, and metabolome, these unidimensional datasets may impart an incomplete or skewed understanding of the underlying factors driving heterogeneity (Comba et al., 2021). New technologies now make it possible to conduct single cell multi-omic analyses to track several molecular features simultaneously allowing researchers to systematically examine the interplay between different sources of heterogeneity and their roles in determining therapeutic response (Vandereyken et al., 2023). In this review, we explore the potential for single cell multi-omic approaches to revolutionize our understanding of how pHGG and other cancers develop treatment resistance and address some of the challenges inherent to applying these technologies towards the development of strategies to improve clinical outcomes in glioma.

## Treatment strategies for pediatric high grade glioma

pHGG is a rare and aggressive form of brain cancer that primarily affects children and adolescents (Ostrom et al., 2022). The discovery of clinically relevant biomarkers through in DNA and RNA sequencing, methylome, and proteomic profiling have led to the recognition of different pHGG subtypes, which are detailed in the 2021 World Health Organization central nervous system tumor classification system (Louis et al., 2021). The importance of establishing prognostic biomarkers for these tumors is exemplified by the diffuse subtypes of pHGG, which may present with similar histological and anatomical features but can exhibit striking differences in their molecular and genetic alterations (Table 1). Fully understanding these distinct molecular and genetic characteristics can then allow clinicians to tailor treatment strategies for pHGG patients based on their tumor subtype (Rallis et al., 2022).

**TABLE 1:** Genetic and molecular characteristics of WHO 2021 classification of pediatric-type diffuse high grade glioma.

Tumor subtype	Molecular features/genetic alterations	Anatomical location	Histological markers	Age at diagnosis	Survival	References
Diffuse midline glioma, H3 K27-altered	H3K27	brainstem, spinal cord, pons, medulla, and thalamus	OLIG2+, H3K27me3 loss, TP53 (variable), ATRX (variable)	7–8 years	9–11 months	Schwartzentruber et al. (2012), Wu et al. (2012), Castel et al. (2015), Cooney et al. (2017)
	TP53					
	ACVR1					
	PDGFRA					
	EGFR					
	EZHIP					
Diffuse hemispheric glioma, H3 G34-mutant	H3G34	supratentorial tumors, mainly in temporal and parietal lobes	GFAP^+^, OLIG2^-^, ATRX^−^, TP53 (variable), MKI67 (high)	15 years	14–18 months	Schwartzentruber et al. (2012), Wu et al. (2012), Korshunov et al. (2016), Lim et al. (2021), Crowell et al. (2022), Gianno et al. (2022)
	TP53					
	ATRX					
Diffuse pediatric-type high-grade glioma, H3-wildtype and IDH-wildtype	IDH-wildtype	supratentorial tumors (∼85%), brainstem (∼15%)	necrosis, microvasculature proliferation, H3K27me3 retained, GFAP^+^, OLIG2^+^	2–18 years	14–44 months	Korshunov et al. (2017), Tauziede-Espariat et al. (2019), Gianno et al. (2022)
	H3-wildtype					
	PDGFRA					
	MYCN					
	EGFR					
Infant-type hemispheric glioma	NTRK family	intra-axial tumor	OLIG2+, GFAP+, H3K27me3 retained	<1 year	5-year overall survival of 50%–60%	Duffner et al. (1999), Guerreiro Stucklin et al. (2019), Ceglie et al. (2020), Clarke et al. (2020), Gianno et al. (2022)
	ALK					
	ROS					
	MET					

Current treatment paradigms for pHGG may vary based on patient age and tumor subtype, but they often include combinations of radiation, chemotherapy, surgery, immunotherapy, and targeted small molecules (Table 2). For some pHGG subtypes, like H3K27-altered tumors and other gliomas with invasive growth patterns, surgical resection is nearly impossible due to risks related to the anatomical locations and the diffuse growth patterns of these tumors (Wang and Jiang, 2013; Jones et al., 2017; Vitanza and Monje, 2019). Radiation is standard of care for pHGG but is ultimately palliative and has minimal impact on overall survival (Mandell et al., 1999; Tauziede-Espariat et al., 2019). Radiotherapy is associated with variable responses in pHGG and can lead to neurocognitive defects especially in very young patients, only increases overall survival in diffuse midline glioma by ∼3 months (Langmoen et al., 1991), and had no significant effect on overall survival for patients diagnosed with H3G34-altered tumors in one recent study (Crowell et al., 2022). Chemotherapy is also rarely effective on its own and leads to variable clinical responses in different in by pHGG subtypes. Treatment with chemotherapy is associated with slightly increased overall survival in infantile and pediatric HGG patients, but exclusively when gross surgical resection was achieved (Wisoff et al., 1998; Wolff et al., 2010). In contrast, multiple studies report that neither TMZ-based nor non-TMZ-based chemotherapy are sufficient to provide a survival benefit for pediatric HGG patients (Spostol et al., 1989; Finlay et al., 1995; Cohen et al., 2011; Jones et al., 2017) and combinations of high dose chemotherapy and radiation similarly did not improve clinical outcomes midline and brainstem tumors or in H3G34-altered tumors (Jenkin et al., 1987; Spostol et al., 1989; Hargrave et al., 2006; Crowell et al., 2022).

**TABLE 2:** Major therapeutic modalities used to treat pediatric high grade glioma.

Therapy category	Examples/sub-categories	References
Radiation	focal radiation, hypo-fractionated radiation	Mandell et al. (1999); Gallitto et al. (2019); Metselaar et al. (2021); Crowell et al. (2022)
Chemotherapy	**Non-TMZ chemotherapy:** CCNU, vincristine, prednisone, methylprednisolone procarbazine, hydroxyurea, cisplatin, cytarabine, dacarbazine	Spostol et al. (1989); Finlay et al. (1995); Duffner et al. (1999); Massimino et al. (2008); Crowell et al. (2022)
	**TMZ chemotherapy**	Lashford et al. (2002); Cohen et al. (2011)
Immunotherapy	T cell immunotherapy	Ahmed et al. (2017); Vitanza et al. (2021b); Haydar et al. (2021); Majzner et al. (2022)
	cancer vaccines	Pollack et al. (2016); Van Gool et al. (2020)
	oncolytic viruses	Friedman et al. (2021); Gallego Perez-Larraya et al. (2022); Ghajar-Rahimi et al. (2022)
	checkpoint inhibitors	Fried et al. (2018); Cacciotti et al. (2020); Van Gool et al. (2020)
	Anti-PD-1 and anti-PD-L1	
Targeted small molecules	panobinostat, vorinostat, ONC-201, dasatinib, crizotinib, everolimus	Arrillaga-Romany et al. (2017); Galanis et al. (2018); Chi et al. (2019); Miklja et al. (2020); Puduvalli et al. (2020); Gibson et al. (2021); DeWire et al. (2022); Izquierdo et al. (2022)

Improved molecular and genetic profiling of pHGG tumors and research in pre-clinical models has led to the discovery of multiple targeted therapies, including both small molecule inhibitors of various enzymes and immunotherapeutic approaches. Targeted small molecule inhibitors for pHGG treatment include: chromatin-targeting drugs like the histone deacetylase inhibitors vorinostat and panobinostat (Fouladi et al., 2010; Jones et al., 2017; Galanis et al., 2018; Puduvalli et al., 2020); the DRD2 antagonist/CLPP agonist ONC201 (Arrillaga-Romany et al., 2017; Chi et al., 2019); kinase inhibitors like dasatinib, crizotinib, and trametinib (Miklja et al., 2020; Gibson et al., 2021; Izquierdo et al., 2022); and mTOR inhibitors like everolimus (Miklja et al., 2020; DeWire et al., 2022). While some of these inhibitors may temporarily improve symptoms and can lead to short-term stable disease in some patients, many of these clinical trials are ongoing and limited in scope, so it is not yet clear whether durable clinical responses will be possible.

More recently, immunotherapeutic approaches using oncolytic viruses, cancer vaccines, and autologous T cell therapy have shown promise in animal models and are under investigation in the clinic. Specifically, clinical trials assessing pHGG patient response to CAR-T cells directed against various tumor antigens, including GD2, B7-H3, and HER2, have led to improved symptoms or stable disease in some patients but have yet to improve overall survival (Ahmed et al., 2017; Vitanza et al., 2021b; Haydar et al., 2021; Majzner et al., 2022). Early phase clinical trials using oncolytic viruses, immune checkpoint inhibitors, and cancer vaccines to target pHGG and enhance tumor immunogenicity are similarly in progress, but it remains uncertain whether these interventions will result in a significant response in larger numbers of patients (Fried et al., 2018; Van Gool et al., 2020; Gallego Perez-Larraya et al., 2022; Ghajar-Rahimi et al., 2022).

In addition to the adverse side effects that can accompany each of these treatment modalities, another factor to consider when developing new therapies is that treatment failure in pHGG commonly occurs due to intrinsic or acquired resistance to therapy (Vanan and Eisenstat, 2014; Kline et al., 2018). Overcoming treatment resistance therefore poses a key challenge to improving clinical outcomes in aggressive and highly heterogeneous cancers like pHGG and will require a better understanding of how resistance to therapy develops in patients. Since subpopulations of brain tumor cells vary in their sensitivity to treatment (Bao et al., 2006; Liu et al., 2006; Chen et al., 2012; Alexander et al., 2020), efforts to elucidate resistance mechanisms in pHGG that rely solely on bulk sample analysis methods or that only focus on a single aspect of tumor cell biology may lose important information regarding changes in sub-clonal tumor cell phenotypes and the tumor microenvironment following treatment. Single cell, multi-omic techniques provide an opportunity to separately profile tumor, normal brain cell, and immune populations (Vandereyken et al., 2023), to characterize dynamic changes to the glioma cellular landscape that occur in response to treatment when applied to pHGG patient samples.

## Single cell multi-omic profiling of glioma

**FIGURE 1:**
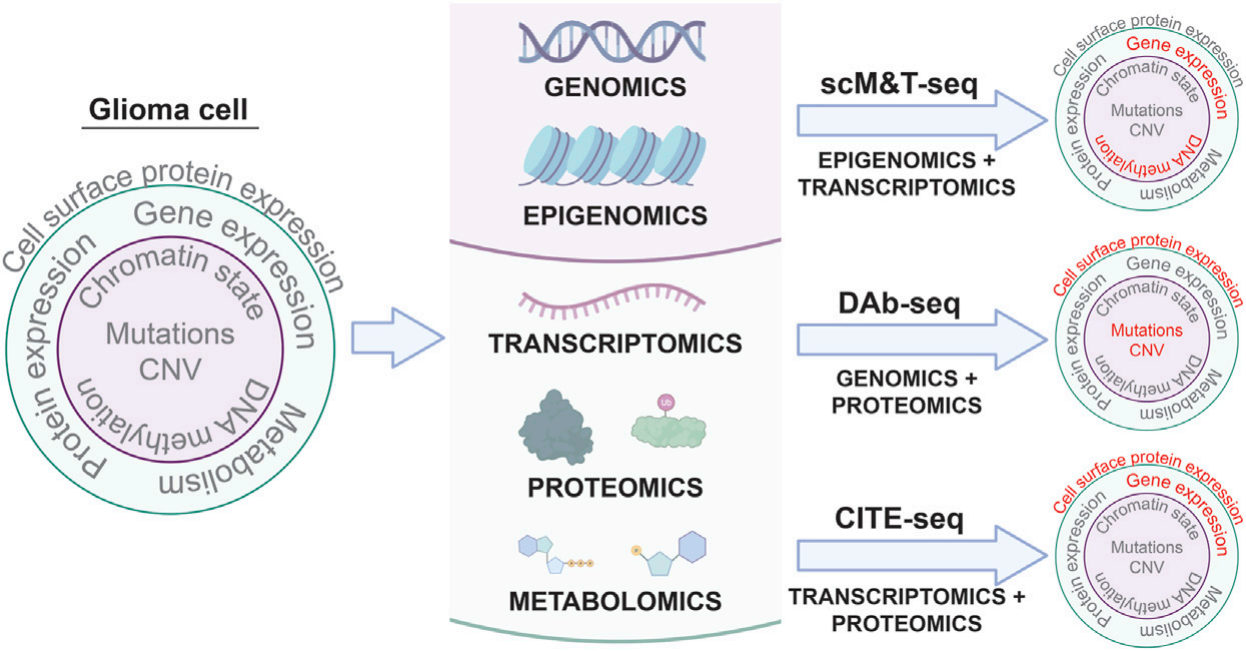
Single cell multi-omic approaches to phenotyping glioma cells. Applying multi-omic techniques to single cells isolated from patient tumors enables simultaneous profiling of different aspects of tumor cell biology in the same cells. Three examples of these types of techniques and the tumor cell phenotypes they characterize are shown: scM&T-seq profiles DNA methylation and gene expression; DAb-seq profiles genomic alterations and cell surface protein expression; and CITE-seq profiles gene expression and cell surface protein expression. Linking multiple phenotypes in individual glioma cells from patient samples through single cell multi-omic approaches like those depicted above has the potential to improve our understanding of intratumoral heterogeneity in glioma and provide insights into the molecular mechanisms driving glioma development and progression.

Differences between the genome, epigenome, transcriptome, proteome, and metabolome of individual tumor cells in a single patient can all contribute to intratumoral heterogeneity associated with therapeutic resistance (Mazor et al., 2016; Marusyk et al., 2020; Santiago et al., 2020). Variable mutation profiles between cells within the same tumor, chromatin dysregulation leading to stem cell-like behaviors in tumor cell sub-populations, neoplastic transcriptional states specific to individual tumor cells, erratic post-translational modifications, and unique metabolic dependencies are all examples of intratumoral differences in cell phenotypes that might influence tumor sensitivity to therapy. Despite the capacity of single cell profiling to reveal mechanisms of tumor heterogeneity and therapeutic resistance, the application of single cell multi-omic analyses to pediatric glioma is rare. As a result, the role of intratumoral heterogeneity in driving pediatric glioma progression and response to treatment is poorly understood. Multi-omic analyses at single-cell resolution can address this knowledge gap by generating more comprehensive profiles of pHGG intratumoral heterogeneity (Figure 1).

Multiple techniques are now available for targeted “omic” profiling of the major subtypes of biological molecules at a single cell resolution (Table 3). Many of these single cell analyses can be performed either as stand-alone assays or in combination with each other to generate multi-omic datasets to describe multiple phenotypic features within single cells, such as through simultaneous analysis of both transcriptomic and epigenetic profiles. For example, combined scRNAseq and single cell ATACseq (scATACseq) has been applied to characterize both single cell transcriptomes and chromatin accessibility patterns in adult spinal ependymoma demonstrating dynamic changes to the landscape of tumor-immune interactions associated with tumor progression (Zhang et al., 2021). The application of both scRNAseq and scATACseq has also recently identified multiple progenitor-like glioma cell subtypes acting cooperatively to maintain tumor growth in adult glioma (Wang et al., 2019), and revealed the complex cellular architectures of different medulloblastoma subtypes (Li et al., 2021).

**TABLE 3:** A brief summary of single cell profiling techniques to assess intratumoral heterogeneity.

Omic data subtypes	Sequencing	Non-sequencing	References
Genome	scDNAseq		Evrony et al. (2021)
Epigenome	scBisulfite-seq, scATACseq, scCUT&RUN/scCUT&Tag		Smallwood et al. (2014); Skene and Henikoff (2017); Kaya-Okur et al. (2019); Xu et al. (2021)
Transcriptome	scRNAseq	MERFISH^a^	Hwang et al. (2018); Xia et al. (2019)
Proteome		Flow cytometry, CyTOF, Imaging mass cytometry^a^, scProteomics	Doxie and Irish (2014); Lavin et al. (2017); Roussel et al. (2020); Woo et al. (2021); Kuett et al. (2022)
Metabolome		SpaceM^a^	Rappez et al. (2021)
Multi-omics			
scG&T-seq	scDNAseq + scRNAseq		Macaulay et al. (2016)
scM&T-seq	scBisulfite-seq + scRNAseq		Angermueller et al. (2016)
scTRIO-seq	scM&T-seq + CNV analysis		Hou et al. (2016)
CITE-seq	cell surface protein + transcriptome profiling by scRNAseq		Peterson et al. (2017); Stoeckius et al. (2017)
REAP-seq			
Watermelon	CITE-seq + lineage tracing		Oren et al. (2021)
SHARE-seq	scRNAseq + scATACseq		Ma et al. (2020); Swanson et al. (2021)
ICICLE-seq			
DAb-seq	cell surface protein + genome profiling by scDNAseq		Morita et al. (2020); Demaree et al. (2021); Peretz et al. (2021)
Tapestri			
Select-seq^a^	RNAseq on SLACS-isolated single cells	+ protein expression by immunofluorescence	Lee et al. (2022)

^a^ = technique preserves spatial information in samples.

Various research groups have also sought to understand the relationship between genetic mutations, DNA methylation and heterogeneous gene expression profiles in glioma patient samples. For example, studies integrating scRNAseq and DNA methylation profiling have uncovered correlations between DNA methylation patterns and gene expression associated with the expression of glial differentiation genes and response to environmental stress (Chaligne et al., 2021; Johnson et al., 2021). An additional study similarly integrated DNA methylation and scRNAseq datasets to identify METTL7B as a potential prognostic marker in glioma associated with an immune-suppressive tumor microenvironment (Chen et al., 2021). Further studies have integrated bulk DNA sequencing and scRNAseq data to track lineage hierarchies and to infer transcriptional signatures associated with distinct glioma sub-populations including stem and progenitor-like cells (Muller et al., 2016; Filbin et al., 2018). Finally, combined genetic profiling, scRNAseq, and metabolic analysis in glioblastoma multiforme has identified new functional subgroups differing in their neurodevelopmental and metabolic profiles that may be targetable in the clinic (Garofano et al., 2021). Together, these studies demonstrate the promise of layering multiple single cell analysis techniques to resolve tumor cell features and to identify functionally significant tumor subpopulations driving resistance.

**FIGURE 2:**
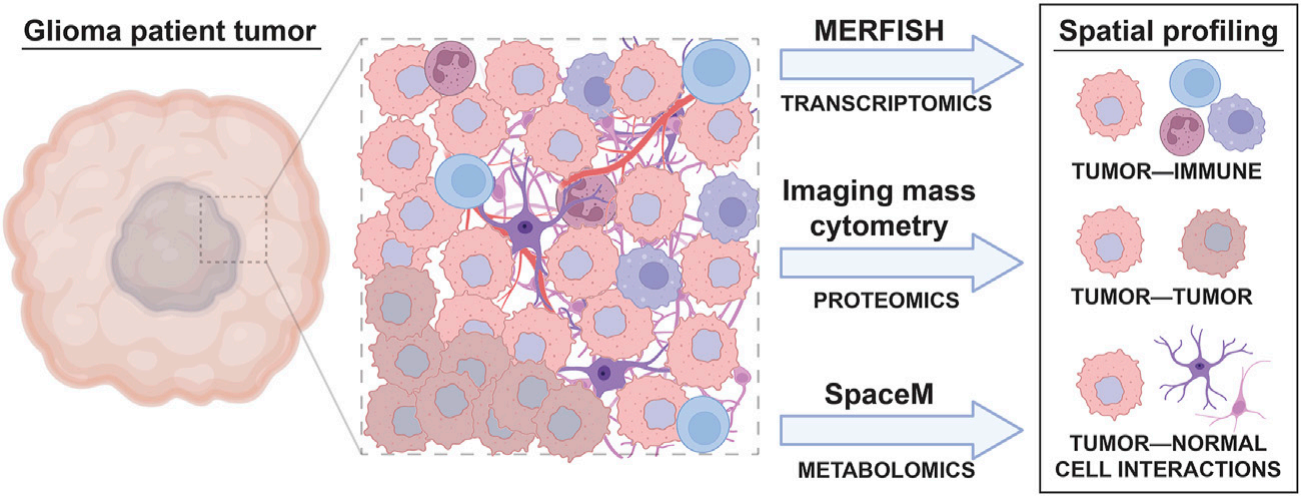
Spatial, single cell profiling of glioma patient tumors. A wide variety of cell types are found in the tumor microenvironment of glioma, including immune cells, different subtypes of tumor cells, and normal brain cells. The interactions between these cells result in tumor cell behavior changes that can be accounted for through spatial, single cell omic profiling techniques. Spatial analyses like MERFISH, imaging mass cytometry, and SpaceM allow the effect of cell to cell interactions on tumor, immune, and normal cell phenotypes to be visualized.

The benefits of using sequencing-based single cell profiling techniques include the generation of extensive amounts of data on large numbers of cells and the ability to simultaneously profile tumor cells, infiltrating immune cells, and normal cells within the same sample. However, one downside of using many single cell sequencing-based techniques is that these analyses usually require cell dissociation as an early step in their sample processing protocols. This need for sample dissociation results in a loss of spatial information regarding the cellular organization of brain tumors, such as local interactions between glioma cells, neurons, normal glia, and immune cells, which may mediate tumor cell phenotypes. For instance, previous studies reveal that glioma stem and progenitor-like cells reside in the perivascular niche in contact with endothelial cells (Calabrese et al., 2007; Charles et al., 2010) and demonstrate extensive spatial heterogeneity in both the abundance and types of immune cells across different regions of brain tumors (Abdelfattah et al., 2022). Conducting spatial profiling of tumor markers through either low throughput (immunohistochemistry, immunocytochemistry, RNA *in situ* analysis) or high throughput (spatial scRNAseq) can therefore complement single cell data from disassociated samples to reveal the regional heterogeneity, and local interactions between, cell types of interest in patient tumors (Figure 2).

## Current knowledge of intratumoral heterogeneity in pediatric high grade glioma

Previous studies have applied single cell multi-omic technologies to characterize intratumoral heterogeneity in glioma, but most reports thus far have focused on adult samples rather than pediatric brain tumors (Table 4). Although a subset of potential therapeutic targets implicated by studies analyzing adult glioma may be relevant to pediatric brain tumors, substantial differences exist in the genetics and molecular features of pHGG (Table 1) that may result in unique mechanisms of therapeutic resistance in pediatric patients (Jones et al., 2012; Sturm et al., 2017; Gestrich et al., 2021; Lehmann et al., 2022). One major difference is that driver mutations in chromatin regulators, including histone H3 point mutations are common in pHGG but not in adult HGG (Khuong-Quang et al., 2012; Schwartzentruber et al., 2012; Wu et al., 2012; Fontebasso et al., 2013; Wiestler et al., 2013; Mackay et al., 2017). The unique biology of pediatric brain tumors may limit the relevance of single cell data from adult samples as a basis to develop therapeutic strategies for childhood glioma and warrants further investigation into the drivers of therapeutic resistance in pHGG.

**TABLE 4:** Selected single cell sequencing datasets from pediatric and adult high grade glioma patient samples.

HGG subtype	Single cell datasets	Major finding	References
Pediatric H3K27M glioma	scRNA-seq	Most H3K27M tumor cells are oligodendrocyte progenitor-like	Filbin et al. (2018)
Pediatric H3.3G34 R/V glioma	scRNA-seq	Epigenetic and transcriptional features of H3.3G34 R/V glioma identify new therapeutic targets	Sweha et al. (2021)
Pediatric cerebellar tumors	scRNA-seq	Tumor cells express fetal transcriptional programs	Vladoiu et al. (2019)
Pediatric ependymoma	scRNA-seq	Neural stem cell-like tumor subclones in relapsed patient samples are more immature with increased immune cell crosstalk	Wu et al. (2022)
Pediatric ependymoma	scRNA-seq	Malignant cell differentiation programs are targetable and predict patient survival	Gojo et al. (2020)
Pediatric medulloblastoma	scRNA-seq	Tumor subtypes with distinct developmental trajectories may share a similar cell-of-origin	Hovestadt et al. (2019)
Pediatric medulloblastoma	scRNA-seq, scATAC-seq	Characterizes medulloblastoma subtypes and identifies potential therapeutic targets	Li et al. (2021)
Adult glioblastoma	scRNA-seq, scATAC-seq	Combination therapy targeting multiple tumor cell phenotypes may improve treatment efficacy	Wang et al. (2019)
Adult glioblastoma	scATAC-seq	An invasive glioma stem cell chromatin state is associated with lower survival	Guilhamon et al. (2021)
Adult glioblastoma	scRNA-seq	The glioma stem cell immune microenvironment transitions from stimulatory to suppressive as tumorigenesis progresses	Zhai et al. (2020)
Adult high grade gliomas	scRNA-seq	Glioma cell subtypes exhibit differences in proliferation and immune cell interactions	Yuan et al. (2018)
Adult glioblastoma	scRNA-seq	Infiltrating immune cells enhance glioblastoma cell proliferation and invasiveness	Darmanis et al. (2017)
Adult glioblastoma	scRNA-seq	Identifies immune suppressive factors in tumor-associated macrophages that can be targeted to improve immunotherapy	Abdelfattah et al. (2022)
Adult glioblastoma	scRNA-seq	Glioblastoma cell states are influenced by DNA mutations and the tumor microenvironment	Neftel et al. (2019)
Adult glioblastoma	scRNA-seq	Individual cells within the same tumor exhibit variable expression of glioblastoma subtype markers	Patel et al. (2014)
Adult glioblastoma	scRNA-seq	Identifies glioma stem cell-specific developmental pathways to target therapeutically	Couturier et al. (2020)
Adult glioblastoma	scRNA-seq, scBisulfite-seq	Epigenetic state can shape therapeutic outcomes	Johnson et al. (2021)
Adult glioblastoma	scRNA-seq	Tumor subtypes exhibit different metabolic dependencies	Garofano et al. (2021)
Adult glioblastoma	scRNA-seq, scBisulfite-seq, scDNA-seq	Malignant cells show epigenetic inheritance that differs based on IDH genotype	Chaligne et al. (2021)

pHGG are not only genetically and molecularly distinct from adult pHGG, but also exhibit a high degree of intra- and intertumoral heterogeneity. Sequencing studies analyzing either multiple brain regions or comparing diagnostic and recurrent tumors taken from individual pHGG patients reveal spatially and temporally heterogeneous DNA mutations even among samples acquired from the same patient affecting genes, such as ATM, PPM1D, BCOR, ATRX, MYC, and KMT5B (Hoffman et al., 2016; Nikbakht et al., 2016; Salloum et al., 2017; Vinci et al., 2018). pHGG also exhibit heterogeneity in the presence of copy number variations in PDGFRA and other genes regulating oncogenic signaling and the cell cycle (Bax et al., 2010; Paugh et al., 2011; Koschmann et al., 2016), and cell to cell variation in epigenetic regulation reflected by differences in histone post-translational modifications and DNA methylation in pHGG (Sturm et al., 2012; Mackay et al., 2017; Castel et al., 2018; Huang et al., 2018). Finally, scRNAseq and histological analyses of both pediatric and adult brain tumors reveal heterogeneous cell phenotypes, including stem and progenitor-like cells as well as neuronal, glial, and mesenchymal subpopulations (Hemmati et al., 2003; Monje et al., 2011; Filbin et al., 2018; Jessa et al., 2019; Vladoiu et al., 2019). Together, these findings suggest that heterogeneity in DNA mutations, epigenetic regulation, transcriptional outputs, and differing tumor microenvironments all have the potential to remodel the cellular landscape of pHGG to promote therapeutic resistance.

Heterogeneity in pHGG can be driven by intrinsic factors like genomic instability and cancer stem cell differentiation as well as in response to extrinsic factors in the tumor microenvironment like tumor-immune cell interactions and drug treatment (Santiago et al., 2020; Kaminska et al., 2021). Multi-omic single cell analyses may therefore identify pathways of previously unknown significance in the development of therapeutic resistance. Single cell multi-omic profiling of brain tumor patient samples have begun to define the complex cellular landscape of adult glioma as it relates to disease biology (Table 4). Recent single cell multi-omic profiling studies have identified salient features of glioma biology, including distinct tumor-immune interactions associated with malignancy and disease progression, correlations between genetic and epigenetic features of tumor cells, novel sub-cellular populations and mechanisms of glioma cell plasticity, and putative therapeutic targets (Wang et al., 2019; Chaligne et al., 2021; Chen et al., 2021; Garofano et al., 2021; Johnson et al., 2021; Zhang et al., 2021). Studies that integrate separate single cell and bulk multi-omic datasets have similarly identified novel regulators, prognostic biomarkers of adult glioma subtypes (Neftel et al., 2019; Wu et al., 2020; Chen et al., 2021). These analyses demonstrate the value of applying single cell, multi-omic profiling as a strategy to reveal hidden complexity in adult gliomas that may soon be applied to pHGG.

In addition to the tumor cell intrinsic factors that drive intratumoral heterogeneity, dynamic changes in tumor composition can occur because of interactions between glioma and immune cells in the surrounding microenvironment (Turner and Reis-Filho, 2012; McGranahan and Swanton, 2017). For example, a single cell transcriptome profiling study in pediatric ependymoma patient samples recently found that neural stem cell-like tumor subclones in relapsed patient samples exhibit increased immune cell crosstalk (Wu et al., 2022). Single cell analysis to reveal dynamic interactions between tumor cells and immune cells may also reveal mechanisms of resistance to immunotherapy and potential targets to enhance pHGG immune targeting. For example, a recent study of pHGG tumors exposed to CAR-T cell therapy using both bulk an single cell profiling revealed heterogeneous CAR-T cell phenotypes and identified cytokines and TGFβ signaling molecules as potential mediators of CAR-T treatment efficacy (Vitanza et al., 2021a; Majzner et al., 2022). Another recent single cell profiling study revealed additional immunosuppressive factors produced by tumor-associated macrophages that might be targeted to enhance therapeutic responses (Abdelfattah et al., 2022).

Intrinsic differences between tumor cells can also set the stage for further, potentially more unpredictable consequences of tumor-tumor cell interactions where clones displaying different phenotypes may have suppressive, protective, or stimulatory effects on each other (Santiago et al., 2020). An example of this behavior comes from a recent paper showing that cells located in distinct anatomical features of glioblastoma multiforme tumors display diverging patterns of gene and protein expression allowing individual tumor cells to influence each other’s behaviors to drive tumor progression and escape from the effects of therapy (Lam et al., 2022). Spatial differences in tumor cell heterogeneity may also influence the activity endogenous immune cells and the efficacy immune cell-based therapies (Liu et al., 2021). Using single cell multi-omic profiling techniques that preserve spatial information in patient samples can therefore provide further insights into the drivers of intratumoral heterogeneity in solid tumors and the effects of a changing tumor microenvironment on treatment response. Despite the valuable knowledge provided by these types of analyses, very little spatial profiling has been done in pHGG. Increasing our knowledge of regional tumor heterogeneity, such as between primary and metastatic tumors invading other brain regions and the spine is particularly needed to understand the diffuse nature of certain subtypes, like DMG which are highly invasive and spread into multiple brain regions (Nikbakht et al., 2016).

**FIGURE 3:**
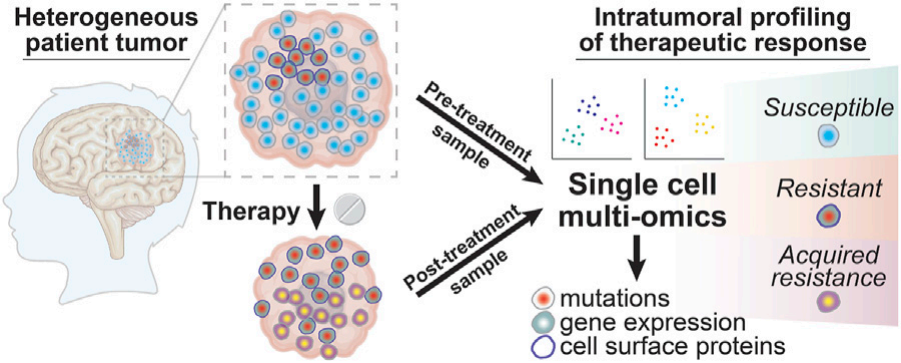
Intratumoral profiling of therapeutic responses in glioma patient tumors with single cell multi-omics. Glioma tumor samples collected from patients before and after treatment can be profiled using single cell multi-omic techniques to identify tumor cell phenotypes associated with successful therapeutic targeting by looking for tumor cell subtypes that are present in pre-treatment samples but that are absent in post-treatment samples. In contrast, resistant tumor cell subtypes will be present in both the pre-treatment and post-treatment sample while newly evolved or adapted tumor cell phenotypes that result in acquired resistance will be seen only in the post-treatment sample. The information yielded from these single cell profiling experiments for tumor cell phenotypes like DNA mutations, gene expression, and cell surface proteins can thus provide a deeper understanding into the clonal composition changes that drive glioma patient responses to different treatment modalities.

These sometimes unpredictable drivers of intratumoral heterogeneity arising from various cell to cell interactions within the tumor microenvironment are perhaps best summarized by the aphorism “the whole is greater than the sum of the parts.” An alternative to bulk analyses is to apply single cell multi-omic analyses to matched glioma patient samples taken before and after treatment to examine the changes in tumor cell phenotypes that occur in response to different forms of therapy (Figure 3). Through well-designed longitudinal studies, it may also be possible to correlate these multi-omic single cell datasets with clinical outcomes as a first step in identifying specific cell populations that escape tumor response or to discover biomarkers of tumor evolution that might predict therapeutic resistance. Analyzing treatment-associated changes in intratumoral heterogeneity in this way will allow researchers to move beyond merely taking the sum of the parts to instead obtain multi-dimensional datasets providing a much more comprehensive view of the pathways and processes underlying therapeutic resistance in brain tumors.

## Profiling treatment-associated changes in the intratumoral heterogeneity in high grade glioma

Standard of care for pHGG consists of radiation often combined with chemotherapy and experimental therapeutics (Fangusaro, 2009; Jones et al., 2012; Adamski et al., 2014; Bredlau and Korones, 2014). Many of these treatments have adverse consequences, including cognitive deficits, endocrine disorders, and vasculopathies, and none of these therapeutic strategies are curative (Armstrong et al., 2004; Merchant et al., 2009; Mueller et al., 2013). pHGG tumors frequently exhibit both intrinsic and acquired resistance to therapy (Vanan and Eisenstat, 2014; Kline et al., 2018) and numerous studies suggest that subpopulations of brain tumor cells vary in their sensitivity to treatment (Bao et al., 2006; Liu et al., 2006; Chen et al., 2012; Alexander et al., 2020). Neither the defining characteristics of treatment-resistant glioma cell sub-populations, nor the underlying pathways regulating spatio-temporal heterogeneity and dynamic response to therapy are fully delineated in pediatric glioma. Our limited knowledge of which genes and pathways drive pediatric glioma heterogeneity and therapeutic resistance represents a key knowledge gap, which has hindered efforts to develop effective treatments to overcome therapeutic resistance in pHGG.

Variable tumor cell phenotypes and mutation profiles can result in different sensitivity to therapy within the same tumor, and the selective pressure applied during treatment can lead to tumor cell adaption and evolution resulting in tumor escape from therapeutic response (Raynaud et al., 2018; Marusyk et al., 2020; Santiago et al., 2020; Touat et al., 2020). Studies comparing matched patient samples collected before and after treatment are more common in cancers like melanoma and leukemia that are more amenable to sample collection compared to central nervous system tumors, and single cell multi-omic profiling of matched samples from these cancers demonstrate that tumor cell evolution correlates with disease relapse and unfavorable treatment outcomes (Gaiti et al., 2019; Granja et al., 2019; Morita et al., 2020). Similar research comparing naïve and treated brain tumor samples has identified features of treatment-resistant glioma either through either bulk or single cell analyses, including potential targets for future therapies, factors that influence the tumor microenvironment, and genomic alterations contributing to malignant progression in different brain tumor subtypes (Barthel et al., 2019; Mueller et al., 2019; Nejo et al., 2019; Petralia et al., 2020). The diverse and impactful findings of these studies emphasize how new technological approaches to profiling intratumoral heterogeneity can provide insight into glioma biology and reveal novel strategies for therapeutic targeting.

Intratumoral heterogeneity observed in pHGG prior to treatment may also result in poor therapeutic response due to the presence of intrinsically resistant tumor cell subpopulations. For example, stem- and progenitor-like glioma cells sometimes known as glioma stem cells or brain tumor initiating cells (Zhou et al., 2021), have been implicated in adult glioma therapeutic response (Bao et al., 2006; Liu et al., 2006; Chen et al., 2012). Recent advances in single cell multi-omic profiling have provided researchers with new tools to decipher roles for rare tumor stem cells in treatment resistance and sensitivity. For example, one study integrated scRNAseq and mass cytometry data from adult glioma samples to identify surface markers used in the isolation of CD9+/CD133+ progenitor cells, which were then shown to be less sensitive to TMZ treatment and more proliferative in intracranial xenografts (Couturier et al., 2020). Another study applied scRNAseq to similarly describe a glioma stem cell subpopulation that was enriched after radiation treatment in a mouse model (Alexander et al., 2020). Additional studies have similarly identified stem cell-like subpopulations in pHGG (Monje et al., 2011; Chen et al., 2012; Filbin et al., 2018), but the role of glioma stem cells in mediating pediatric glioma phenotypes and response to therapy has not yet been thoroughly studied. However, there is some evidence that glioma stem cells may play a role in pHGG recurrence after therapy (Hoffman et al., 2019). Further studies applying single cell multi-omic analyses to pHGG patient tumors before and after therapy may help unravel potential roles for cancer stem cells and other glioma cell sub-populations in both intrinsic and acquired resistance.

## Validating results from multi-omic single cell datasets using mouse models

A limitation of many single cell profiling studies is that these analyses generate correlative data, and the functional relevance of many molecular markers and cell sub-populations revealed by these analyses can remain elusive. Single cell analyses of mouse models of pHGG may also provide valuable insights into the underlying causes of tumor heterogeneity and reveal mechanisms of treatment resistance. Pairing patient tumor molecular profiling data with results from functional biological assays in cell culture and animal models is one strategy for experimentally determining the mechanisms of tumor heterogeneity and treatment resistance. For example, recent studies have sought to analyze differences in gene expression and chromatin accessibility in isolated adult glioma stem cell clones varying in their proliferation, differentiation, and sensitivity to TMZ to identify gene regulatory signatures associated with these aggressive tumor cell phenotypes (Meyer et al., 2015; Guilhamon et al., 2021). By subsequently comparing these gene and chromatin signatures to single cell datasets from patient tumors, the authors then provided evidence that freshly dissociated tumors contain cells exhibiting similar characteristics to the aggressive clones profiled in the laboratory, highlighting the potential significance of these phenotypes. Another study performed both molecular profiling and drug screening in newly established primary cell culture models derived from patient tumor samples collected in an ongoing clinical trial to identify the mechanism of resistance by which DMG tumors evade MEK inhibitors (Izquierdo et al., 2022). In this study, MEK inhibitor resistance occurred through the development of *de novo* mutations that might serve as therapeutic targets in a combination treatment strategy to ablate the resistant tumor cells. This approach combining molecular profiling and functional drug testing has the potential to identify other promising therapies if applied to additional treatment modalities, particularly if the functional studies are accompanied by single cell multi-omic profiling of pre- and post-treatment pHGG patient samples.

Comparative single cell profiling of patient samples and mouse models also has the potential to yield significant insights into therapeutic resistance and the roles of tumor-immune cell interactions in glioma treatment response. Of note, a recent comparative single cell multi-omic study in adult glioblastoma found conserved changes in tumor-associated immune cell responses to therapy between mouse models and patient tumors, suggesting that animal studies have the potential to accurately recapitulate certain aspects of patient tumor microenvironment (Pombo Antunes et al., 2021). Given that glioblastoma driver mutations are known to influence the immune composition of the tumor microenvironment in adult HGG (Garcia-Fabiani et al., 2021), additional comparative studies using single cell multi-omic techniques to analyze recently developed immunocompetent models of pHGG may therefore provide insight into the effects of patient tumor subtype on therapy resistance due to immunosuppressive or immunostimulatory states (Chen and Hambardzumyan, 2018; Patel et al., 2020; Tomita et al., 2022). Together, these studies emphasize how combining data from multi-omic single cell profiling of patient samples and results obtained from preclinical model systems may help researchers to better understand the significance of specific molecular profiles on glioma cell behaviors like drug resistance or response to immunotherapy.

## Challenges and conclusion

A practical concern regarding the use of multi-omic single cell sequencing approaches to study glioma disease mechanisms is that these experiments generate massive data outputs requiring complex analysis pipelines and extensive resources for data storage. In contrast, single cell profiling by non-sequencing approaches may be more cost-effective and can potentially preserve tissue structures to provide an additional layer of information about the tumor microenvironment and cell to cell interactions (Figure 2). Low-throughput and non-sequencing based approaches, like immunohistochemistry and DNA fluorescence *in situ* hybridization (FISH), can offer much faster, cheaper, and readily interpretable assessments of tumor heterogeneity while still assaying large numbers of cells. However, the information provided will be more biased than sequencing-based single cell profiling since non-sequencing techniques rely on a relatively small number of probes or antibodies, which may result in more limited findings from exploratory studies (Kashyap et al., 2022).

Given the wide range of single cell profiling techniques (Table 1), the high cost associated with sequencing-based single cell analyses, and the limited availability of pHGG patient samples, selecting among potential methods for single cell profiling requires careful consideration. For example, some single cell profiling techniques can be performed on fixed tissue, while others require fresh or frozen specimens. Appropriate single cell profiling methodologies must also be selected according to the treatment modalities in question. Single cell analyses that conserve spatial information may be particularly useful for characterizing glioma responses to immunotherapy because these methods enable the visualization of tumor-immune interactions (Marusyk et al., 2020). Since immunotherapy is beginning to show some promising results in early stage clinical trials for pHGG (Majzner et al., 2022), profiling pre- and post-treatment patient tumor samples using spatial, single cell multi-omic techniques may be especially relevant to ongoing efforts to improve immunotherapeutic approaches for pHGG treatment.

Single cell multi-omic datasets can also be expensive to generate and require processing through complex analytical pipelines before the results can be interpreted. The types of bioinformatic analyses and mathematical modeling that are applied to single cell multi-omic datasets are a key determinant of the quality of information obtained from these experiments. Unfortunately, bioinformatic analysis protocols for single cell datasets are not yet standardized across different research studies. Depending on the pipelines used, in-depth analyses of multiple aspects of intratumoral heterogeneity can be made from the shared properties identified between single cell multi-omic datasets or even extracted from individual single cell omic profiling experiments, such as by detecting somatic mutations or multi-cellular programs from scRNAseq data (Vu et al., 2019; Welch et al., 2019; Jerby-Arnon and Regev, 2022). Separate analyses using mathematical modeling of treatment efficacy can then take this intratumoral heterogeneity into account in simulations aimed at predicting determinants of patient responses to therapy (Rockne and Scott, 2019). However, bioinformatic analyses and mathematical modeling have their own limitations and may also provide an incomplete or inaccurate understanding of how tumor heterogeneity contributes to therapy resistance. Further, integrating single cell datasets generated from separate experiments or even from different studies through meta-analyses may also yield important findings despite the inability to examine different “omics” datasets in the same cells.

Perhaps the most daunting challenge limiting current studies applying single cell multi-omic analyses to naïve and treatment-resistant brain tumors is the ability of researchers to access paired tumor samples across the time course of a patient’s treatment. Matched single cell analyses in non-responding or relapsed patients either during surgical resection procedures or from rapid autopsies may provide a viable strategy to study the development of therapeutic resistance that arises from intratumoral heterogeneity in glioma. Using pre-treatment patient biopsies to generate organoid, explant, and gliomasphere cultures or patient-derived orthotopic xenograft mice can also allow for pre-treatment and post-treatment responses to be assessed without requiring multiple rounds of brain tumor sample collection from a single patient (Hubert et al., 2016; Jacob et al., 2020; LeBlanc et al., 2022; Sundar et al., 2022). However, these *in vitro* and *in vivo* patient-derived glioma models may not accurately reflect the effects of the intratumoral heterogeneity and the tumor microenvironment on treatment resistance. Primary patient samples collected before and after treatment are therefore better suited for use in single cell multi-omic studies even though paired patient brain tumor sample collection can prove challenging. Despite these limitations, the recent introduction of lower-cost sequencing technology coupled with the rising use of machine learning to assist with data processing and interpretation have made single cell multi-omic profiling easier than ever and greatly improved its potential to yield clinically significant data when applied to patient samples.

## Future directions for assessing intratumoral heterogeneity in glioma

Resistance to therapies, such as radiation, chemotherapy, targeted therapeutics, and immunotherapy, is a key challenge to improving clinical outcomes in aggressive and highly heterogeneous cancers like pHGG, which have no effective treatment options despite decades of research. The development of therapeutic resistance in these tumors is a multifaceted process that may be attributed to: 1) the expansion of subclonal populations showing intrinsic resistance; 2) the emergence of altered tumor cell phenotypes as these cells adapt or evolve upon exposure to therapy and escape killing; and 3) dynamic changes to the tumor microenvironment, including the interactions between brain tumor cells and normal neurons, glia, and tumor-associated immune cells. Before the advent of single cell technologies, previous research into the molecular underpinnings of therapeutic resistance relied on bulk approaches that assessed molecular changes induced by treatment at low resolution and averaged across large numbers of cells without the ability to separately profile tumor, non-tumor, and immune populations. In this review, we have highlighted how sophisticated, new technologies now enable researchers to layer multi-dimensional datasets to simultaneously profile genetic, epigenetic, transcriptomic, and proteomic aberrations at a single cell level to describe dynamic changes to the glioma cellular landscape in response to treatment.

Most of the existing single cell multi-omic analyses of glioma published thus far have focused on either determining how tumor cell phenotypes diverge from normal tissue or on sub-classifying tumors into different molecular subgroups. Applying single cell multi-omic profiling to pre- and post-treatment pHGG tumors has the potential to reveal the consequences of various therapies on tumor composition and to identify markers of treatment susceptibility and resistance. These types of analyses are expected to generate data that can be applied to ultimately improve patient outcomes in several ways: by identifying novel treatment strategies and combination therapies to target the mechanisms by which glioma escapes therapy, by predicting the outcomes of different treatment modalities in a patient-specific manner that accounts for heterogeneity in therapeutic responses between cells in the same tumor, and by assessing how tumor-immune interactions affect response to therapy and resistance. Obtaining a more holistic view of the complex interplay between diverse tumor cell populations and their environment through multi-omic single cell analyses provides an opportunity to revolutionize therapeutic approaches to cancer treatment and make precision oncology practice more precise.

## References

[B1] Abdelfattah N. Kumar P. Wang C. Leu J. S. Flynn W. F. Gao R. (2022). Single-cell analysis of human glioma and immune cells identifies S100A4 as an immunotherapy target. Nat. Commun. 13, 767. 10.1038/s41467-022-28372-y 35140215 PMC8828877

[B2] Adamski J. Tabori U. Bouffet E. (2014). Advances in the management of paediatric high-grade glioma. Curr. Oncol. Rep. 16, 414. 10.1007/s11912-014-0414-0 25410415

[B3] Ahmed N. Brawley V. Hegde M. Bielamowicz K. Kalra M. Landi D. (2017). HER2-Specific chimeric antigen receptor-modified virus-specific T cells for progressive glioblastoma: A phase 1 dose-escalation trial. JAMA Oncol. 3, 1094–1101. 10.1001/jamaoncol.2017.0184 28426845 PMC5747970

[B4] Alexander J. Laplant Q. C. Pattwell S. S. Szulzewsky F. Cimino P. J. Caruso F. P. (2020). Multimodal single-cell analysis reveals distinct radioresistant stem-like and progenitor cell populations in murine glioma. Glia 68, 2486–2502. 10.1002/glia.23866 32621641 PMC7586969

[B5] Allen C. E. Laetsch T. W. Mody R. Irwin M. S. Lim M. S. Adamson P. C. (2017). Target and agent prioritization for the children's oncology group-national cancer institute pediatric MATCH trial. J. Natl. Cancer Inst. 109, djw274. 10.1093/jnci/djw274 28376230 PMC5963793

[B6] Angermueller C. Clark S. J. Lee H. J. Macaulay I. C. Teng M. J. Hu T. X. (2016). Parallel single-cell sequencing links transcriptional and epigenetic heterogeneity. Nat. Methods 13, 229–232. 10.1038/nmeth.3728 26752769 PMC4770512

[B7] Armstrong C. L. Gyato K. Awadalla A. W. Lustig R. Tochner Z. A. (2004). A critical review of the clinical effects of therapeutic irradiation damage to the brain: The roots of controversy. Neuropsychol. Rev. 14, 65–86. 10.1023/b:nerv.0000026649.68781.8e 15260139

[B8] Arrillaga-Romany I. Chi A. S. Allen J. E. Oster W. Wen P. Y. Batchelor T. T. (2017). A phase 2 study of the first imipridone ONC201, a selective DRD2 antagonist for oncology, administered every three weeks in recurrent glioblastoma. Oncotarget 8, 79298–79304. 10.18632/oncotarget.17837 29108308 PMC5668041

[B9] Bao S. Wu Q. Mclendon R. E. Hao Y. Shi Q. Hjelmeland A. B. (2006). Glioma stem cells promote radioresistance by preferential activation of the DNA damage response. Nature 444, 756–760. 10.1038/nature05236 17051156

[B10] Barthel F. P. Johnson K. C. Varn F. S. Moskalik A. D. Tanner G. Kocakavuk E. (2019). Longitudinal molecular trajectories of diffuse glioma in adults. Nature 576, 112–120. 10.1038/s41586-019-1775-1 31748746 PMC6897368

[B11] Bax D. A. Mackay A. Little S. E. Carvalho D. Viana-Pereira M. Tamber N. (2010). A distinct spectrum of copy number aberrations in pediatric high-grade gliomas. Clin. Cancer Res. 16, 3368–3377. 10.1158/1078-0432.CCR-10-0438 20570930 PMC2896553

[B12] Bredlau A. L. Korones D. N. (2014). Diffuse intrinsic pontine gliomas: Treatments and controversies. Adv. Cancer Res. 121, 235–259. 10.1016/B978-0-12-800249-0.00006-8 24889533

[B13] Cacciotti C. Choi J. Alexandrescu S. Zimmerman M. A. Cooney T. M. Chordas C. (2020). Immune checkpoint inhibition for pediatric patients with recurrent/refractory CNS tumors: A single institution experience. J. Neurooncol 149, 113–122. 10.1007/s11060-020-03578-6 32627129

[B14] Calabrese C. Poppleton H. Kocak M. Hogg T. L. Fuller C. Hamner B. (2007). A perivascular niche for brain tumor stem cells. Cancer Cell 11, 69–82. 10.1016/j.ccr.2006.11.020 17222791

[B15] Castel D. Philippe C. Calmon R. Le Dret L. Truffaux N. Boddaert N. (2015). Histone H3F3A and HIST1H3B K27M mutations define two subgroups of diffuse intrinsic pontine gliomas with different prognosis and phenotypes. Acta Neuropathol. 130, 815–827. 10.1007/s00401-015-1478-0 26399631 PMC4654747

[B16] Castel D. Philippe C. Kergrohen T. Sill M. Merlevede J. Barret E. (2018). Transcriptomic and epigenetic profiling of 'diffuse midline gliomas, H3 K27M-mutant' discriminate two subgroups based on the type of histone H3 mutated and not supratentorial or infratentorial location. Acta Neuropathol. Commun. 6, 117. 10.1186/s40478-018-0614-1 30396367 PMC6219253

[B17] Ceglie G. Vinci M. Carai A. Rossi S. Colafati G. S. Cacchione A. (2020). Infantile/congenital high-grade gliomas: Molecular features and therapeutic perspectives. Diagn. (Basel) 10, 648. 10.3390/diagnostics10090648 PMC7555400 32872331

[B18] Chaligne R. Gaiti F. Silverbush D. Schiffman J. S. Weisman H. R. Kluegel L. (2021). Epigenetic encoding, heritability and plasticity of glioma transcriptional cell states. Nat. Genet. 53, 1469–1479. 10.1038/s41588-021-00927-7 34594037 PMC8675181

[B19] Charles N. Ozawa T. Squatrito M. Bleau A. M. Brennan C. W. Hambardzumyan D. (2010). Perivascular nitric oxide activates notch signaling and promotes stem-like character in PDGF-induced glioma cells. Cell Stem Cell 6, 141–152. 10.1016/j.stem.2010.01.001 20144787 PMC3818090

[B20] Chen J. Li Y. Yu T. S. Mckay R. M. Burns D. K. Kernie S. G. (2012). A restricted cell population propagates glioblastoma growth after chemotherapy. Nature 488, 522–526. 10.1038/nature11287 22854781 PMC3427400

[B21] Chen X. Li C. Li Y. Wu S. Liu W. Lin T. (2021). Characterization of METTL7B to evaluate TME and predict prognosis by integrative analysis of multi-omics data in glioma. Front. Mol. Biosci. 8, 727481. 10.3389/fmolb.2021.727481 34604305 PMC8484875

[B22] Chen Z. Hambardzumyan D. (2018). Immune microenvironment in glioblastoma subtypes. Front. Immunol. 9, 1004. 10.3389/fimmu.2018.01004 29867979 PMC5951930

[B23] Chi A. S. Tarapore R. S. Hall M. D. Shonka N. Gardner S. Umemura Y. (2019). Pediatric and adult H3 K27M-mutant diffuse midline glioma treated with the selective DRD2 antagonist ONC201. J. Neurooncol 145, 97–105. 10.1007/s11060-019-03271-3 31456142 PMC7241441

[B24] Clarke M. Mackay A. Ismer B. Pickles J. C. Tatevossian R. G. Newman S. (2020). Infant high-grade gliomas comprise multiple subgroups characterized by novel targetable gene fusions and favorable outcomes. Cancer Discov. 10, 942–963. 10.1158/2159-8290.CD-19-1030 32238360 PMC8313225

[B25] Cohen A. R. (2022). Brain tumors in children. N. Engl. J. Med. 386, 1922–1931. 10.1056/NEJMra2116344 35584157

[B26] Cohen K. J. Pollack I. F. Zhou T. Buxton A. Holmes E. J. Burger P. C. (2011). Temozolomide in the treatment of high-grade gliomas in children: A report from the children's oncology group. Neuro Oncol. 13, 317–323. 10.1093/neuonc/noq191 21339192 PMC3064602

[B27] Comba A. Faisal S. M. Varela M. L. Hollon T. Al-Holou W. N. Umemura Y. (2021). Uncovering spatiotemporal heterogeneity of high-grade gliomas: From disease biology to therapeutic implications. Front. Oncol. 11, 703764. 10.3389/fonc.2021.703764 34422657 PMC8377724

[B28] Cooney T. Lane A. Bartels U. Bouffet E. Goldman S. Leary S. E. S. (2017). Contemporary survival endpoints: An international diffuse intrinsic pontine glioma registry study. Neuro Oncol. 19, 1279–1280. 10.1093/neuonc/nox107 28821206 PMC5570207

[B29] Couturier C. P. Ayyadhury S. Le P. U. Nadaf J. Monlong J. Riva G. (2020). Single-cell RNA-seq reveals that glioblastoma recapitulates a normal neurodevelopmental hierarchy. Nat. Commun. 11, 3406. 10.1038/s41467-020-17186-5 32641768 PMC7343844

[B30] Crowell C. Mata-Mbemba D. Bennett J. Matheson K. Mackley M. Perreault S. (2022). Systematic review of diffuse hemispheric glioma, H3 G34-mutant: Outcomes and associated clinical factors. Neurooncol Adv. 4, vdac133. 10.1093/noajnl/vdac133 36105387 PMC9466272

[B31] Curry R. N. Aiba I. Meyer J. Lozzi B. Ko Y. Mcdonald M. F. (2023). Glioma epileptiform activity and progression are driven by IGSF3-mediated potassium dysregulation. Neuron 111, 682–695 e9. 10.1016/j.neuron.2023.01.013 36787748 PMC9991983

[B32] Darmanis S. Sloan S. A. Croote D. Mignardi M. Chernikova S. Samghababi P. (2017). Single-cell RNA-seq analysis of infiltrating neoplastic cells at the migrating front of human glioblastoma. Cell Rep. 21, 1399–1410. 10.1016/j.celrep.2017.10.030 29091775 PMC5810554

[B33] Demaree B. Delley C. L. Vasudevan H. N. Peretz C. A. C. Ruff D. Smith C. C. (2021). Joint profiling of DNA and proteins in single cells to dissect genotype-phenotype associations in leukemia. Nat. Commun. 12, 1583. 10.1038/s41467-021-21810-3 33707421 PMC7952600

[B34] Dewire M. Lazow M. Campagne O. Leach J. Fuller C. Senthil Kumar S. (2022). Phase I study of ribociclib and everolimus in children with newly diagnosed DIPG and high-grade glioma: A connect pediatric neuro-oncology consortium report. Neurooncol Adv. 4, vdac055. vdac055. 10.1093/noajnl/vdac055 35611273 PMC9122788

[B35] Doxie D. B. Irish J. M. (2014). High-dimensional single-cell cancer biology. Curr. Top. Microbiol. Immunol. 377, 1–21. 10.1007/82_2014_367 24671264 PMC4216808

[B36] Duffner P. K. Horowitz M. E. Krischer J. P. Burger P. C. Cohen M. E. Sanford R. A. (1999). The treatment of malignant brain tumors in infants and very youngchildren: An update of the pediatric oncology group experience. Neuro Oncol. 1, 152–161. 10.1093/neuonc/1.2.152 11554387 PMC1920752

[B37] Evrony G. D. Hinch A. G. Luo C. (2021). Applications of single-cell DNA sequencing. Annu. Rev. Genomics Hum. Genet. 22, 171–197. 10.1146/annurev-genom-111320-090436 33722077 PMC8410678

[B38] Fangusaro J. (2009). Pediatric high-grade gliomas and diffuse intrinsic pontine gliomas. J. Child Neurology 24, 1409–1417. 10.1177/0883073809338960 19638636

[B39] Filbin M. G. Tirosh I. Hovestadt V. Shaw M. L. Escalante L. E. Mathewson N. D. (2018). Developmental and oncogenic programs in H3K27M gliomas dissected by single-cell RNA-seq. Science 360, 331–335. 10.1126/science.aao4750 29674595 PMC5949869

[B40] Finlay J. L. Boyett J. M. Yates A. J. Wisoff J. H. Milstein J. M. Geyer J. R. (1995). Randomized phase III trial in childhood high-grade astrocytoma comparing vincristine, lomustine, and prednisone with the eight-drugs-in-1-day regimen. Childrens cancer group. J. Clin. Oncol. 13, 112–123. 10.1200/JCO.1995.13.1.112 7799011

[B41] Fontebasso A. M. Schwartzentruber J. Khuong-Quang D. A. Liu X. Y. Sturm D. Korshunov A. (2013). Mutations in SETD2 and genes affecting histone H3K36 methylation target hemispheric high-grade gliomas. Acta Neuropathol. 125, 659–669. 10.1007/s00401-013-1095-8 23417712 PMC3631313

[B42] Fouladi M. Park J. R. Stewart C. F. Gilbertson R. J. Schaiquevich P. Sun J. (2010). Pediatric phase I trial and pharmacokinetic study of vorinostat: A children's oncology group phase I consortium report. J. Clin. Oncol. 28, 3623–3629. 10.1200/JCO.2009.25.9119 20606092 PMC2917318

[B43] Fried I. Lossos A. Ben Ami T. Dvir R. Toledano H. Ben Arush M. W. (2018). Preliminary results of immune modulating antibody MDV9300 (pidilizumab) treatment in children with diffuse intrinsic pontine glioma. J. Neurooncol 136, 189–195. 10.1007/s11060-017-2643-1 29143272

[B44] Friedman G. K. Johnston J. M. Bag A. K. Bernstock J. D. Li R. Aban I. (2021). Oncolytic HSV-1 G207 immunovirotherapy for pediatric high-grade gliomas. N. Engl. J. Med. 384, 1613–1622. 10.1056/NEJMoa2024947 33838625 PMC8284840

[B45] Gaiti F. Chaligne R. Gu H. Brand R. M. Kothen-Hill S. Schulman R. C. (2019). Epigenetic evolution and lineage histories of chronic lymphocytic leukaemia. Nature 569, 576–580. 10.1038/s41586-019-1198-z 31092926 PMC6533116

[B46] Galanis E. Anderson S. K. Miller C. R. Sarkaria J. N. Jaeckle K. Buckner J. C. (2018). Phase I/II trial of vorinostat combined with temozolomide and radiation therapy for newly diagnosed glioblastoma: Results of alliance N0874/ABTC 02. Neuro Oncol. 20, 546–556. 10.1093/neuonc/nox161 29016887 PMC5909661

[B47] Gallego Perez-Larraya J. Garcia-Moure M. Labiano S. Patino-Garcia A. Dobbs J. Gonzalez-Huarriz M. (2022). Oncolytic DNX-2401 virus for pediatric diffuse intrinsic pontine glioma. N. Engl. J. Med. 386, 2471–2481. 10.1056/NEJMoa2202028 35767439

[B48] Gallitto M. Lazarev S. Wasserman I. Stafford J. M. Wolden S. L. Terezakis S. A. (2019). Role of radiation therapy in the management of diffuse intrinsic pontine glioma: A systematic review. Adv. Radiat. Oncol. 4, 520–531. 10.1016/j.adro.2019.03.009 31360809 PMC6639749

[B49] Garcia-Fabiani M. B. Haase S. Comba A. Carney S. Mcclellan B. Banerjee K. (2021). Genetic alterations in gliomas remodel the tumor immune microenvironment and impact immune-mediated therapies. Front. Oncol. 11, 631037. 10.3389/fonc.2021.631037 34168976 PMC8217836

[B50] Garofano L. Migliozzi S. Oh Y. T. D'Angelo F. Najac R. D. Ko A. (2021). Pathway-based classification of glioblastoma uncovers a mitochondrial subtype with therapeutic vulnerabilities. Nat. Cancer 2, 141–156. 10.1038/s43018-020-00159-4 33681822 PMC7935068

[B51] Gestrich C. K. Jajosky A. N. Elliott R. Stearns D. Sadri N. Cohen M. L. (2021). Molecular profiling of pediatric and adult glioblastoma. Am. J. Clin. Pathol. 155, 606–614. 10.1093/ajcp/aqaa172 33210143

[B52] Ghajar-Rahimi G. Kang K. D. Totsch S. K. Gary S. Rocco A. Blitz S. (2022). Clinical advances in oncolytic virotherapy for pediatric brain tumors. Pharmacol. Ther. 239, 108193. 10.1016/j.pharmthera.2022.108193 35487285 PMC9709696

[B53] Gianno F. Giovannoni I. Cafferata B. Diomedi-Camassei F. Minasi S. Barresi S. (2022). Paediatric-type diffuse high-grade gliomas in the 5th CNS WHO Classification. Pathologica 114, 422–435. 10.32074/1591-951X-830 36534421 PMC9763979

[B54] Gibson E. G. Campagne O. Selvo N. S. Gajjar A. Stewart C. F. (2021). Population pharmacokinetic analysis of crizotinib in children with progressive/recurrent high-grade and diffuse intrinsic pontine gliomas. Cancer Chemother. Pharmacol. 88, 1009–1020. 10.1007/s00280-021-04357-4 34586478 PMC8561710

[B55] Gojo J. Englinger B. Jiang L. Hubner J. M. Shaw M. L. Hack O. A. (2020). Single-cell RNA-seq reveals cellular hierarchies and impaired developmental trajectories in pediatric ependymoma. Cancer Cell 38, 44–59. 10.1016/j.ccell.2020.06.004 32663469 PMC7479515

[B56] Granja J. M. Klemm S. Mcginnis L. M. Kathiria A. S. Mezger A. Corces M. R. (2019). Single-cell multiomic analysis identifies regulatory programs in mixed-phenotype acute leukemia. Nat. Biotechnol. 37, 1458–1465. 10.1038/s41587-019-0332-7 31792411 PMC7258684

[B57] Guerreiro Stucklin A. S. Ryall S. Fukuoka K. Zapotocky M. Lassaletta A. Li C. (2019). Alterations in ALK/ROS1/NTRK/MET drive a group of infantile hemispheric gliomas. Nat. Commun. 10, 4343. 10.1038/s41467-019-12187-5 31554817 PMC6761184

[B58] Guilhamon P. Chesnelong C. Kushida M. M. Nikolic A. Singhal D. Macleod G. (2021). Single-cell chromatin accessibility profiling of glioblastoma identifies an invasive cancer stem cell population associated with lower survival. Elife 10, e64090. 10.7554/eLife.64090 33427645 PMC7847307

[B59] Hargrave D. Bartels U. Bouffet E. (2006). Diffuse brainstem glioma in children: Critical review of clinical trials. Lancet Oncol. 7, 241–248. 10.1016/S1470-2045(06)70615-5 16510333

[B60] Haydar D. Houke H. Chiang J. Yi Z. Ode Z. Caldwell K. (2021). Cell-surface antigen profiling of pediatric brain tumors: B7-H3 is consistently expressed and can be targeted via local or systemic CAR T-cell delivery. Neuro Oncol. 23, 999–1011. 10.1093/neuonc/noaa278 33320196 PMC8168826

[B61] Hemmati H. D. Nakano I. Lazareff J. A. Masterman-Smith M. Geschwind D. H. Bronner-Fraser M. (2003). Cancerous stem cells can arise from pediatric brain tumors. Proc. Natl. Acad. Sci. U. S. A. 100, 15178–15183. 10.1073/pnas.2036535100 14645703 PMC299944

[B62] Hoffman L. M. Dewire M. Ryall S. Buczkowicz P. Leach J. Miles L. (2016). Spatial genomic heterogeneity in diffuse intrinsic pontine and midline high-grade glioma: Implications for diagnostic biopsy and targeted therapeutics. Acta Neuropathol. Commun. 4, 1. 10.1186/s40478-015-0269-0 26727948 PMC4700584

[B63] Hoffman M. Gillmor A. H. Kunz D. J. Johnston M. J. Nikolic A. Narta K. (2019). Intratumoral genetic and functional heterogeneity in pediatric glioblastoma. Cancer Res. 79, 2111–2123. 10.1158/0008-5472.CAN-18-3441 30877103 PMC7282886

[B64] Hou Y. Guo H. Cao C. Li X. Hu B. Zhu P. (2016). Single-cell triple omics sequencing reveals genetic, epigenetic, and transcriptomic heterogeneity in hepatocellular carcinomas. Cell Res. 26, 304–319. 10.1038/cr.2016.23 26902283 PMC4783472

[B65] Hovestadt V. Smith K. S. Bihannic L. Filbin M. G. Shaw M. L. Baumgartner A. (2019). Resolving medulloblastoma cellular architecture by single-cell genomics. Nature 572, 74–79. 10.1038/s41586-019-1434-6 31341285 PMC6754173

[B66] Huang T. Garcia R. Qi J. Lulla R. Horbinski C. Behdad A. (2018). Detection of histone H3 K27M mutation and post-translational modifications in pediatric diffuse midline glioma via tissue immunohistochemistry informs diagnosis and clinical outcomes. Oncotarget 9, 37112–37124. 10.18632/oncotarget.26430 30647848 PMC6324678

[B67] Hubert C. G. Rivera M. Spangler L. C. Wu Q. Mack S. C. Prager B. C. (2016). A three-dimensional organoid culture system derived from human glioblastomas recapitulates the hypoxic gradients and cancer stem cell heterogeneity of tumors found *in vivo* . Cancer Res. 76, 2465–2477. 10.1158/0008-5472.CAN-15-2402 26896279 PMC4873351

[B68] Hwang B. Lee J. H. Bang D. (2018). Single-cell RNA sequencing technologies and bioinformatics pipelines. Exp. Mol. Med. 50, 96–14. 10.1038/s12276-018-0071-8 30089861 PMC6082860

[B69] ICGC PEDBRAIN-SEQ PROJECT ICGC MMML-SEQ PROJECT, Grobner S. N. Worst B. C. Weischenfeldt J. Buchhalter I. (2018). The landscape of genomic alterations across childhood cancers. Nature 555, 321–327. 10.1038/nature25480 29489754

[B70] Izquierdo E. Carvalho D. M. Mackay A. Temelso S. Boult J. K. R. Pericoli G. (2022). DIPG harbors alterations targetable by MEK inhibitors, with acquired resistance mechanisms overcome by combinatorial inhibition. Cancer Discov. 12, 712–729. 10.1158/2159-8290.CD-20-0930 34737188 PMC7612484

[B71] Jacob F. Salinas R. D. Zhang D. Y. Nguyen P. T. T. Schnoll J. G. Wong S. Z. H. (2020). A patient-derived glioblastoma organoid model and biobank recapitulates inter- and intra-tumoral heterogeneity. Cell 180, 188–204. 10.1016/j.cell.2019.11.036 31883794 PMC7556703

[B72] Jenkin R. D. T. Boesel C. Ertel I. Evans A. Hittle T. R. Ortega J. (1987). Brain-stem tumors in childhood: A prospective randomized trial of irradiation with and without adjuvant CCNU, vcr, and prednisone: A report of the childrens cancer study group. J. Neurosurg. 66, 227–233. 10.3171/jns.1987.66.2.0227 3806204

[B73] Jerby-Arnon L. Regev A. (2022). DIALOGUE maps multicellular programs in tissue from single-cell or spatial transcriptomics data. Nat. Biotechnol. 40, 1467–1477. 10.1038/s41587-022-01288-0 35513526 PMC9547813

[B74] Jessa S. Blanchet-Cohen A. Krug B. Vladoiu M. Coutelier M. Faury D. (2019). Stalled developmental programs at the root of pediatric brain tumors. Nat. Genet. 51, 1702–1713. 10.1038/s41588-019-0531-7 31768071 PMC6885128

[B75] Johnson K. C. Anderson K. J. Courtois E. T. Gujar A. D. Barthel F. P. Varn F. S. (2021). Single-cell multimodal glioma analyses identify epigenetic regulators of cellular plasticity and environmental stress response. Nat. Genet. 53, 1456–1468. 10.1038/s41588-021-00926-8 34594038 PMC8570135

[B76] Jones C. Karajannis M. A. Jones D. T. W. Kieran M. W. Monje M. Baker S. J. (2017). Pediatric high-grade glioma: Biologically and clinically in need of new thinking. Neuro Oncol. 19, 153–161. 10.1093/neuonc/now101 27282398 PMC5464243

[B77] Jones C. Perryman L. Hargrave D. (2012). Paediatric and adult malignant glioma: Close relatives or distant cousins? Nat. Rev. Clin. Oncol. 9, 400–413. 10.1038/nrclinonc.2012.87 22641364

[B78] Kaminska B. Ochocka N. Segit P. (2021). Single-cell omics in dissecting immune microenvironment of malignant gliomas-challenges and perspectives. Cells 10, 2264. 10.3390/cells10092264 34571910 PMC8470971

[B79] Kashyap A. Rapsomaniki M. A. Barros V. Fomitcheva-Khartchenko A. Martinelli A. L. Rodriguez A. F. (2022). Quantification of tumor heterogeneity: From data acquisition to metric generation. Trends Biotechnol. 40, 647–676. 10.1016/j.tibtech.2021.11.006 34972597

[B80] Kaya-Okur H. S. Wu S. J. Codomo C. A. Pledger E. S. Bryson T. D. Henikoff J. G. (2019). CUT&Tag for efficient epigenomic profiling of small samples and single cells. Nat. Commun. 10, 1930. 10.1038/s41467-019-09982-5 31036827 PMC6488672

[B81] Khan A. B. Lee S. Harmanci A. S. Patel R. Latha K. Yang Y. (2023). CXCR4 expression is associated with proneural-to-mesenchymal transition in glioblastoma. Int. J. Cancer 152, 713–724. 10.1002/ijc.34329 36250346 PMC10071545

[B82] Khuong-Quang D. A. Buczkowicz P. Rakopoulos P. Liu X. Y. Fontebasso A. M. Bouffet E. (2012). K27M mutation in histone H3.3 defines clinically and biologically distinct subgroups of pediatric diffuse intrinsic pontine gliomas. Acta Neuropathol. 124, 439–447. 10.1007/s00401-012-0998-0 22661320 PMC3422615

[B83] Kline C. Felton E. Allen I. E. Tahir P. Mueller S. (2018). Survival outcomes in pediatric recurrent high-grade glioma: Results of a 20-year systematic review and meta-analysis. J. Neurooncol 137, 103–110. 10.1007/s11060-017-2701-8 29204840 PMC5823744

[B84] Korshunov A. Capper D. Reuss D. Schrimpf D. Ryzhova M. Hovestadt V. (2016). Histologically distinct neuroepithelial tumors with histone 3 G34 mutation are molecularly similar and comprise a single nosologic entity. Acta Neuropathol. 131, 137–146. 10.1007/s00401-015-1493-1 26482474

[B85] Korshunov A. Schrimpf D. Ryzhova M. Sturm D. Chavez L. Hovestadt V. (2017). H3-/IDH-wild type pediatric glioblastoma is comprised of molecularly and prognostically distinct subtypes with associated oncogenic drivers. Acta Neuropathol. 134, 507–516. 10.1007/s00401-017-1710-1 28401334

[B86] Koschmann C. Zamler D. Mackay A. Robinson D. Wu Y. Doherty R. (2016). Characterizing and targeting PDGFRA alterations in pediatric high-grade glioma. Oncotarget 7, 65696–65706. 10.18632/oncotarget.11602 27582545 PMC5323185

[B87] Kuett L. Catena R. Ozcan A. Pluss A. Cancer Grand Challenges I. C. Schraml P. (2022). Three-dimensional imaging mass cytometry for highly multiplexed molecular and cellular mapping of tissues and the tumor microenvironment. Nat. Cancer 3, 122–133. 10.1038/s43018-021-00301-w 35121992 PMC7613779

[B88] Lam K. H. B. Leon A. J. Hui W. Lee S. C. Batruch I. Faust K. (2022). Topographic mapping of the glioblastoma proteome reveals a triple-axis model of intra-tumoral heterogeneity. Nat. Commun. 13, 116. 10.1038/s41467-021-27667-w 35013227 PMC8748638

[B89] Langmoen I. A. Lundar T. Storm-Mathisen I. Lie S. O. Hovind K. H. (1991). Management of pediatric pontine gliomas. J. Int. Soc. Pediatr. Neurosurg. 7, 13–15. 10.1007/BF00263825 2054800

[B90] Lashford L. S. Thiesse P. Jouvet A. Jaspan T. Couanet D. Griffiths P. D. (2002). Temozolomide in malignant gliomas of childhood: A United Kingdom children's cancer study group and French society for pediatric oncology intergroup study. J. Clin. Oncol. 20, 4684–4691. 10.1200/JCO.2002.08.141 12488414

[B91] Lavin Y. Kobayashi S. Leader A. Amir E. D. Elefant N. Bigenwald C. (2017). Innate immune landscape in early lung adenocarcinoma by paired single-cell analyses. Cell 169, 750–765. 10.1016/j.cell.2017.04.014 28475900 PMC5737939

[B92] Leblanc V. G. Trinh D. L. Aslanpour S. Hughes M. Livingstone D. Jin D. (2022). Single-cell landscapes of primary glioblastomas and matched explants and cell lines show variable retention of inter- and intratumor heterogeneity. Cancer Cell 40, 379–392 e9. 10.1016/j.ccell.2022.02.016 35303420

[B93] Lee A. C. Lee Y. Choi A. Lee H. B. Shin K. Lee H. (2022). Spatial epitranscriptomics reveals A-to-I editome specific to cancer stem cell microniches. Nat. Commun. 13, 2540. 10.1038/s41467-022-30299-3 35534484 PMC9085828

[B94] Lehmann R. Rayner B. S. Ziegler D. S. (2022). Resistance mechanisms in BRAF(V600E) paediatric high-grade glioma and current therapeutic approaches. Front. Oncol. 12, 1031378. 10.3389/fonc.2022.1031378 36582791 PMC9792688

[B95] Li Z. Wei Y. Shao Y. Tang L. Gong J. (2021). Multi-omics analysis of intertumoral heterogeneity within medulloblastoma uncharted-pathway subtypes. Brain Tumor Pathol. 38, 234–242. 10.1007/s10014-021-00400-7 34180021

[B96] Lim K. Y. Won J. K. Park C. K. Kim S. K. Choi S. H. Kim T. (2021). H3 G34-mutant high-grade glioma. Brain Tumor Pathol. 38, 4–13. 10.1007/s10014-020-00378-8 32995948

[B97] Liu G. Yuan X. Zeng Z. Tunici P. Ng H. Abdulkadir I. R. (2006). Analysis of gene expression and chemoresistance of CD133+ cancer stem cells in glioblastoma. Mol. Cancer 5, 67. 10.1186/1476-4598-5-67 17140455 PMC1697823

[B98] Liu J. Qu S. Zhang T. Gao Y. Shi H. Song K. (2021). Applications of single-cell omics in tumor Immunology. Front. Immunol. 12, 697412. 10.3389/fimmu.2021.697412 34177965 PMC8221107

[B99] Louis D. N. Perry A. Wesseling P. Brat D. J. Cree I. A. Figarella-Branger D. (2021). The 2021 WHO classification of tumors of the central nervous system: A summary. Neuro Oncol. 23, 1231–1251. 10.1093/neuonc/noab106 34185076 PMC8328013

[B100] Ma S. Zhang B. Lafave L. M. Earl A. S. Chiang Z. Hu Y. (2020). Chromatin potential identified by shared single-cell profiling of RNA and chromatin. Cell 183, 1103–1116. 10.1016/j.cell.2020.09.056 33098772 PMC7669735

[B101] Ma X. Liu Y. Liu Y. Alexandrov L. B. Edmonson M. N. Gawad C. (2018). Pan-cancer genome and transcriptome analyses of 1,699 paediatric leukaemias and solid tumours. Nature 555, 371–376. 10.1038/nature25795 29489755 PMC5854542

[B102] Macaulay I. C. Teng M. J. Haerty W. Kumar P. Ponting C. P. Voet T. (2016). Separation and parallel sequencing of the genomes and transcriptomes of single cells using G&T-seq. Nat. Protoc. 11, 2081–2103. 10.1038/nprot.2016.138 27685099

[B103] Mackay A. Burford A. Carvalho D. Izquierdo E. Fazal-Salom J. Taylor K. R. (2017). Integrated molecular meta-analysis of 1,000 pediatric high-grade and diffuse intrinsic pontine glioma. Cancer Cell 32, 520–537. 10.1016/j.ccell.2017.08.017 28966033 PMC5637314

[B104] Majzner R. G. Ramakrishna S. Yeom K. W. Patel S. Chinnasamy H. Schultz L. M. (2022). GD2-CAR T cell therapy for H3K27M-mutated diffuse midline gliomas. Nature 603, 934–941. 10.1038/s41586-022-04489-4 35130560 PMC8967714

[B105] Mandell L. R. Kadota R. Freeman C. Douglass E. C. Fontanesi J. Cohen M. E. (1999). There is No role for hyperfractionated radiotherapy in the management of children with newly diagnosed diffuse intrinsic brainstem tumors: Results of a pediatric oncology group phase III trial comparing conventional vs. Hyperfractionated radiotherapy. Int. J. Radiat. Oncol. Biol. Phys. 43, 959–964. 10.1016/s0360-3016(98)00501-x 10192340

[B106] Marusyk A. Janiszewska M. Polyak K. (2020). Intratumor heterogeneity: The rosetta stone of therapy resistance. Cancer Cell 37, 471–484. 10.1016/j.ccell.2020.03.007 32289271 PMC7181408

[B107] Massimino M. Spreafico F. Biassoni V. Simonetti F. Riva D. Trecate G. (2008). Diffuse pontine gliomas in children: Changing strategies, changing results? A mono-institutional 20-year experience. J. Neurooncol 87, 355–361. 10.1007/s11060-008-9525-5 18217208

[B108] Mazor T. Pankov A. Song J. S. Costello J. F. (2016). Intratumoral heterogeneity of the epigenome. Cancer Cell 29, 440–451. 10.1016/j.ccell.2016.03.009 27070699 PMC4852161

[B109] Mcgranahan N. Swanton C. (2017). Clonal heterogeneity and tumor evolution: Past, present, and the future. Cell 168, 613–628. 10.1016/j.cell.2017.01.018 28187284

[B110] Merchant T. E. Conklin H. M. Wu S. Lustig R. H. Xiong X. (2009). Late effects of conformal radiation therapy for pediatric patients with low-grade glioma: Prospective evaluation of cognitive, endocrine, and hearing deficits. J. Clin. Oncol. 27, 3691–3697. 10.1200/JCO.2008.21.2738 19581535 PMC2799064

[B111] Metselaar D. S. Du Chatinier A. Stuiver I. Kaspers G. J. L. Hulleman E. (2021). Radiosensitization in pediatric high-grade glioma: Targets, resistance and developments. Front. Oncol. 11, 662209. 10.3389/fonc.2021.662209 33869066 PMC8047603

[B112] Meyer M. Reimand J. Lan X. Head R. Zhu X. Kushida M. (2015). Single cell-derived clonal analysis of human glioblastoma links functional and genomic heterogeneity. Proc. Natl. Acad. Sci. U. S. A. 112, 851–856. 10.1073/pnas.1320611111 25561528 PMC4311802

[B113] Miklja Z. Yadav V. N. Cartaxo R. T. Siada R. Thomas C. C. Cummings J. R. (2020). Everolimus improves the efficacy of dasatinib in PDGFRα-driven glioma. J. Clin. Invest. 130, 5313–5325. 10.1172/JCI133310 32603316 PMC7524471

[B114] Monje M. Mitra S. S. Freret M. E. Raveh T. B. Kim J. Masek M. (2011). Hedgehog-responsive candidate cell of origin for diffuse intrinsic pontine glioma. Proc. Natl. Acad. Sci. U. S. A. 108, 4453–4458. 10.1073/pnas.1101657108 21368213 PMC3060250

[B115] Morita K. Wang F. Jahn K. Hu T. Tanaka T. Sasaki Y. (2020). Clonal evolution of acute myeloid leukemia revealed by high-throughput single-cell genomics. Nat. Commun. 11, 5327. 10.1038/s41467-020-19119-8 33087716 PMC7577981

[B116] Mueller S. Fullerton H. J. Stratton K. Leisenring W. Weathers R. E. Stovall M. (2013). Radiation, atherosclerotic risk factors, and stroke risk in survivors of pediatric cancer: A report from the childhood cancer survivor study. Int. J. Radiat. Oncol. Biol. Phys. 86, 649–655. 10.1016/j.ijrobp.2013.03.034 23680033 PMC3696633

[B117] Mueller S. Jain P. Liang W. S. Kilburn L. Kline C. Gupta N. (2019). A pilot precision medicine trial for children with diffuse intrinsic pontine glioma-pnoc003: A report from the pacific pediatric neuro-oncology consortium. Int. J. Cancer 145, 1889–1901. 10.1002/ijc.32258 30861105

[B118] Muller S. Liu S. J. Di Lullo E. Malatesta M. Pollen A. A. Nowakowski T. J. (2016). Single-cell sequencing maps gene expression to mutational phylogenies in PDGF- and EGF-driven gliomas. Mol. Syst. Biol. 12, 889. 10.15252/msb.20166969 27888226 PMC5147052

[B119] Neftel C. Laffy J. Filbin M. G. Hara T. Shore M. E. Rahme G. J. (2019). An integrative model of cellular states, plasticity, and genetics for glioblastoma. Cell 178, 835–849. 10.1016/j.cell.2019.06.024 31327527 PMC6703186

[B120] Nejo T. Matsushita H. Karasaki T. Nomura M. Saito K. Tanaka S. (2019). Reduced neoantigen expression revealed by longitudinal multiomics as a possible immune evasion mechanism in glioma. Cancer Immunol. Res. 7, 1148–1161. 10.1158/2326-6066.CIR-18-0599 31088845

[B121] Nikbakht H. Panditharatna E. Mikael L. G. Li R. Gayden T. Osmond M. (2016). Spatial and temporal homogeneity of driver mutations in diffuse intrinsic pontine glioma. Nat. Commun. 7, 11185. 10.1038/ncomms11185 27048880 PMC4823825

[B122] Oren Y. Tsabar M. Cuoco M. S. Amir-Zilberstein L. Cabanos H. F. Hutter J. C. (2021). Cycling cancer persister cells arise from lineages with distinct programs. Nature 596, 576–582. 10.1038/s41586-021-03796-6 34381210 PMC9209846

[B123] Ostrom Q. T. Cioffi G. Gittleman H. Patil N. Waite K. Kruchko C. (2019). CBTRUS statistical report: Primary brain and other central nervous system tumors diagnosed in the United States in 2012-2016. Neuro Oncol. 21, v1–v100. 10.1093/neuonc/noz150 31675094 PMC6823730

[B124] Ostrom Q. T. Price M. Neff C. Cioffi G. Waite K. A. Kruchko C. (2022). CBTRUS statistical report: Primary brain and other central nervous system tumors diagnosed in the United States in 2015-2019. Neuro Oncol. 24, v1–v95. 10.1093/neuonc/noac202 36196752 PMC9533228

[B125] Patel A. P. Tirosh I. Trombetta J. J. Shalek A. K. Gillespie S. M. Wakimoto H. (2014). Single-cell RNA-seq highlights intratumoral heterogeneity in primary glioblastoma. Science 344, 1396–1401. 10.1126/science.1254257 24925914 PMC4123637

[B126] Patel S. K. Hartley R. M. Wei X. Furnish R. Escobar-Riquelme F. Bear H. (2020). Generation of diffuse intrinsic pontine glioma mouse models by brainstem-targeted *in utero* electroporation. Neuro Oncol. 22, 381–392. 10.1093/neuonc/noz197 31638150 PMC7442382

[B127] Paugh B. S. Broniscer A. Qu C. Miller C. P. Zhang J. Tatevossian R. G. (2011). Genome-wide analyses identify recurrent amplifications of receptor tyrosine kinases and cell-cycle regulatory genes in diffuse intrinsic pontine glioma. J. Clin. Oncol. 29, 3999–4006. 10.1200/JCO.2011.35.5677 21931021 PMC3209696

[B128] Peretz C. A. C. Mcgary L. H. F. Kumar T. Jackson H. Jacob J. Durruthy-Durruthy R. (2021). Single-cell DNA sequencing reveals complex mechanisms of resistance to quizartinib. Blood Adv. 5, 1437–1441. 10.1182/bloodadvances.2020003398 33666651 PMC7948271

[B129] Peterson V. M. Zhang K. X. Kumar N. Wong J. Li L. Wilson D. C. (2017). Multiplexed quantification of proteins and transcripts in single cells. Nat. Biotechnol. 35, 936–939. 10.1038/nbt.3973 28854175

[B130] Petralia F. Tignor N. Reva B. Koptyra M. Chowdhury S. Rykunov D. (2020). Integrated proteogenomic characterization across major histological types of pediatric brain cancer. Cell 183, 1962–1985 e31. 10.1016/j.cell.2020.10.044 33242424 PMC8143193

[B131] Pollack I. F. Jakacki R. I. Butterfield L. H. Hamilton R. L. Panigrahy A. Normolle D. P. (2016). Antigen-specific immunoreactivity and clinical outcome following vaccination with glioma-associated antigen peptides in children with recurrent high-grade gliomas: Results of a pilot study. J. Neurooncol 130, 517–527. 10.1007/s11060-016-2245-3 27624914 PMC5363717

[B132] Pombo Antunes A. R. Scheyltjens I. Lodi F. Messiaen J. Antoranz A. Duerinck J. (2021). Single-cell profiling of myeloid cells in glioblastoma across species and disease stage reveals macrophage competition and specialization. Nat. Neurosci. 24, 595–610. 10.1038/s41593-020-00789-y 33782623

[B133] Puduvalli V. K. Wu J. Yuan Y. Armstrong T. S. Vera E. Wu J. (2020). A Bayesian adaptive randomized phase II multicenter trial of bevacizumab with or without vorinostat in adults with recurrent glioblastoma. Neuro Oncol. 22, 1505–1515. 10.1093/neuonc/noaa062 32166308 PMC7686463

[B134] Rallis K. S. George A. M. Wozniak A. M. Bigogno C. M. Chow B. Hanrahan J. G. (2022). Molecular genetics and targeted therapies for paediatric high-grade glioma. Cancer Genomics Proteomics 19, 390–414. 10.21873/cgp.20328 35732328 PMC9247880

[B135] Rappez L. Stadler M. Triana S. Gathungu R. M. Ovchinnikova K. Phapale P. (2021). SpaceM reveals metabolic states of single cells. Nat. Methods 18, 799–805. 10.1038/s41592-021-01198-0 34226721 PMC7611214

[B136] Raynaud F. Mina M. Tavernari D. Ciriello G. (2018). Pan-cancer inference of intra-tumor heterogeneity reveals associations with different forms of genomic instability. PLoS Genet. 14, e1007669. 10.1371/journal.pgen.1007669 30212491 PMC6155543

[B137] Rockne R. C. Scott J. G. (2019). Introduction to mathematical oncology. JCO Clin. Cancer Inf. 3, 1–4. 10.1200/CCI.19.00010 PMC6752950 31026176

[B138] Roussel M. Lhomme F. Roe C. E. Bartkowiak T. Gravelle P. Laurent C. (2020). Mass cytometry defines distinct immune profile in germinal center B-cell lymphomas. Cancer Immunol. Immunother. 69, 407–420. 10.1007/s00262-019-02464-z 31919622 PMC7764565

[B139] Salloum R. Mcconechy M. K. Mikael L. G. Fuller C. Drissi R. Dewire M. (2017). Characterizing temporal genomic heterogeneity in pediatric high-grade gliomas. Acta Neuropathol. Commun. 5, 78. 10.1186/s40478-017-0479-8 29084603 PMC5663045

[B140] Santiago R. Y. C. Sese M. Capdevila C. Aasen T. De Mattos-Arruda L. Diaz-Cano S. J. (2020). Clinical implications of intratumor heterogeneity: Challenges and opportunities. J. Mol. Med. Berl. 98, 161–177. 10.1007/s00109-020-01874-2 31970428 PMC7007907

[B141] Schwartzentruber J. Korshunov A. Liu X. Y. Jones D. T. Pfaff E. Jacob K. (2012). Driver mutations in histone H3.3 and chromatin remodelling genes in paediatric glioblastoma. Nature 482, 226–231. 10.1038/nature10833 22286061

[B142] Skene P. J. Henikoff S. (2017). An efficient targeted nuclease strategy for high-resolution mapping of DNA binding sites. Elife 6, e21856. 10.7554/eLife.21856 28079019 PMC5310842

[B143] Smallwood S. A. Lee H. J. Angermueller C. Krueger F. Saadeh H. Peat J. (2014). Single-cell genome-wide bisulfite sequencing for assessing epigenetic heterogeneity. Nat. Methods 11, 817–820. 10.1038/nmeth.3035 25042786 PMC4117646

[B144] Sottoriva A. Spiteri I. Piccirillo S. G. Touloumis A. Collins V. P. Marioni J. C. (2013). Intratumor heterogeneity in human glioblastoma reflects cancer evolutionary dynamics. Proc. Natl. Acad. Sci. U. S. A. 110, 4009–4014. 10.1073/pnas.1219747110 23412337 PMC3593922

[B145] Spostol R. Ertel I. J. Jenkin R. D. T. Boesel C. P. Venes J. L. Ortega J. A. (1989). The effectiveness of chemotherapy for treatment of high grade astrocytoma in children: Results of a randomized trial: A report from the childrens cancer study group. J. Neurooncol 7, 165–177. 10.1007/bf00165101 2550594

[B146] Stoeckius M. Hafemeister C. Stephenson W. Houck-Loomis B. Chattopadhyay P. K. Swerdlow H. (2017). Simultaneous epitope and transcriptome measurement in single cells. Nat. Methods 14, 865–868. 10.1038/nmeth.4380 28759029 PMC5669064

[B147] Sturm D. Pfister S. M. Jones D. T. W. (2017). Pediatric gliomas: Current concepts on diagnosis, biology, and clinical management. J. Clin. Oncol. 35, 2370–2377. 10.1200/JCO.2017.73.0242 28640698

[B148] Sturm D. Witt H. Hovestadt V. Khuong-Quang D. A. Jones D. T. Konermann C. (2012). Hotspot mutations in H3F3A and IDH1 define distinct epigenetic and biological subgroups of glioblastoma. Cancer Cell 22, 425–437. 10.1016/j.ccr.2012.08.024 23079654

[B149] Sundar S. J. Shakya S. Barnett A. Wallace L. C. Jeon H. Sloan A. (2022). Three-dimensional organoid culture unveils resistance to clinical therapies in adult and pediatric glioblastoma. Transl. Oncol. 15, 101251. 10.1016/j.tranon.2021.101251 34700192 PMC8551697

[B150] Swanson E. Lord C. Reading J. Heubeck A. T. Genge P. C. Thomson Z. (2021). Simultaneous trimodal single-cell measurement of transcripts, epitopes, and chromatin accessibility using TEA-seq. Elife 10, e63632. 10.7554/eLife.63632 33835024 PMC8034981

[B151] Sweha S. R. Chung C. Natarajan S. K. Panwalkar P. Pun M. Ghali A. (2021). Epigenetically defined therapeutic targeting in H3.3G34R/V high-grade gliomas. Sci. Transl. Med. 13, eabf7860. 10.1126/scitranslmed.abf7860 34644147 PMC8783551

[B152] Syafruddin S. E. Nazarie W. Moidu N. A. Soon B. H. Mohtar M. A. (2021). Integration of RNA-Seq and proteomics data identifies glioblastoma multiforme surfaceome signature. BMC Cancer 21, 850. 10.1186/s12885-021-08591-0 34301218 PMC8306276

[B153] Tauziede-Espariat A. Debily M. A. Castel D. Grill J. Puget S. Sabel M. (2019). An integrative radiological, histopathological and molecular analysis of pediatric pontine histone-wildtype glioma with MYCN amplification (HGG-MYCN). Acta Neuropathol. Commun. 7, 87. 10.1186/s40478-019-0738-y 31177990 PMC6556947

[B154] Tomita Y. Shimazu Y. Somasundaram A. Tanaka Y. Takata N. Ishi Y. (2022). A novel mouse model of diffuse midline glioma initiated in neonatal oligodendrocyte progenitor cells highlights cell-of-origin dependent effects of H3K27M. Glia 70, 1681–1698. 10.1002/glia.24189 35524725 PMC9546478

[B155] Touat M. Li Y. Y. Boynton A. N. Spurr L. F. Iorgulescu J. B. Bohrson C. L. (2020). Mechanisms and therapeutic implications of hypermutation in gliomas. Nature 580, 517–523. 10.1038/s41586-020-2209-9 32322066 PMC8235024

[B156] Turner N. C. Reis-Filho J. S. (2012). Genetic heterogeneity and cancer drug resistance. Lancet Oncol. 13, e178–e185. 10.1016/S1470-2045(11)70335-7 22469128

[B157] Van Gool S. W. Makalowski J. Bonner E. R. Feyen O. Domogalla M. P. Prix L. (2020). Addition of multimodal immunotherapy to combination treatment strategies for children with DIPG: A single institution experience. Med. (Basel) 7, 29. 10.3390/medicines7050029 PMC7281768 32438648

[B158] Vanan M. I. Eisenstat D. D. (2014). Management of high-grade gliomas in the pediatric patient: Past, present, and future. Neurooncol Pract. 1, 145–157. 10.1093/nop/npu022 26034626 PMC4369714

[B159] Vandereyken K. Sifrim A. Thienpont B. Voet T. (2023). Methods and applications for single-cell and spatial multi-omics. Nat. Rev. Genet. 2023, 1–22. 10.1038/s41576-023-00580-2 PMC9979144 36864178

[B160] Vinci M. Burford A. Molinari V. Kessler K. Popov S. Clarke M. (2018). Functional diversity and cooperativity between subclonal populations of pediatric glioblastoma and diffuse intrinsic pontine glioma cells. Nat. Med. 24, 1204–1215. 10.1038/s41591-018-0086-7 29967352 PMC6086334

[B161] Vitanza N. A. Biery M. C. Myers C. Ferguson E. Zheng Y. Girard E. J. (2021a). Optimal therapeutic targeting by HDAC inhibition in biopsy-derived treatment-naive diffuse midline glioma models. Neuro Oncol. 23, 376–386. 10.1093/neuonc/noaa249 33130903 PMC7992886

[B162] Vitanza N. A. Johnson A. J. Wilson A. L. Brown C. Yokoyama J. K. Kunkele A. (2021b). Locoregional infusion of HER2-specific CAR T cells in children and young adults with recurrent or refractory CNS tumors: An interim analysis. Nat. Med. 27, 1544–1552. 10.1038/s41591-021-01404-8 34253928

[B163] Vitanza N. A. Monje M. (2019). Diffuse intrinsic pontine glioma: From diagnosis to next-generation clinical trials. Curr. Treat. Options Neurol. 21, 37. 10.1007/s11940-019-0577-y 31290035 PMC10234769

[B164] Vladoiu M. C. El-Hamamy I. Donovan L. K. Farooq H. Holgado B. L. Sundaravadanam Y. (2019). Childhood cerebellar tumours mirror conserved fetal transcriptional programs. Nature 572, 67–73. 10.1038/s41586-019-1158-7 31043743 PMC6675628

[B165] Vu T. N. Nguyen H. N. Calza S. Kalari K. R. Wang L. Pawitan Y. (2019). Cell-level somatic mutation detection from single-cell RNA sequencing. Bioinformatics 35, 4679–4687. 10.1093/bioinformatics/btz288 31028395 PMC6853710

[B166] Wang L. Babikir H. Muller S. Yagnik G. Shamardani K. Catalan F. (2019). The phenotypes of proliferating glioblastoma cells reside on a single Axis of variation. Cancer Discov. 9, 1708–1719. 10.1158/2159-8290.CD-19-0329 31554641 PMC7161589

[B167] Wang Y. Jiang T. (2013). Understanding high grade glioma: Molecular mechanism, therapy and comprehensive management. Cancer Lett. 331, 139–146. 10.1016/j.canlet.2012.12.024 23340179

[B168] Wang Z. Wang Y. Yang T. Xing H. Wang Y. Gao L. (2021). Prediction of RBP binding sites on circRNAs using an LSTM-based deep sequence learning architecture. Brief. Bioinform 22, bbab342. 10.1093/bib/bbab342 34415289

[B169] Welch J. D. Kozareva V. Ferreira A. Vanderburg C. Martin C. Macosko E. Z. (2019). Single-cell multi-omic integration compares and contrasts features of brain cell identity. Cell 177, 1873–1887. 10.1016/j.cell.2019.05.006 31178122 PMC6716797

[B170] Wen P. Y. Kesari S. (2008). Malignant gliomas in adults. N. Engl. J. Med. 359, 492–507. 10.1056/NEJMra0708126 18669428

[B171] Wiestler B. Capper D. Holland-Letz T. Korshunov A. Von Deimling A. Pfister S. M. (2013). ATRX loss refines the classification of anaplastic gliomas and identifies a subgroup of IDH mutant astrocytic tumors with better prognosis. Acta Neuropathol. 126, 443–451. 10.1007/s00401-013-1156-z 23904111

[B172] Wisoff J. H. Boyett J. M. Berger M. S. Brant C. Li H. Yates A. J. (1998). Current neurosurgical management and the impact of the extent of resection in the treatment of malignant gliomas of childhood: A report of the children’s cancer group trial No. CCG-945. J. Neurosurg. 89, 52–59. 10.3171/jns.1998.89.1.0052 9647172

[B173] Wolff J. E. Driever P. H. Erdlenbruch B. Kortmann R. D. Rutkowski S. Pietsch T. (2010). Intensive chemotherapy improves survival in pediatric high-grade glioma after gross total resection: Results of the HIT-GBM-C protocol. Cancer 116, 705–712. 10.1002/cncr.24730 19957326

[B174] Woo J. Williams S. M. Markillie L. M. Feng S. Tsai C. F. Aguilera-Vazquez V. (2021). High-throughput and high-efficiency sample preparation for single-cell proteomics using a nested nanowell chip. Nat. Commun. 12, 6246. 10.1038/s41467-021-26514-2 34716329 PMC8556371

[B175] Wu G. Broniscer A. Mceachron T. A. Lu C. Paugh B. S. Becksfort J. (2012). Somatic histone H3 alterations in pediatric diffuse intrinsic pontine gliomas and non-brainstem glioblastomas. Nat. Genet. 44, 251–253. 10.1038/ng.1102 22286216 PMC3288377

[B176] Wu H. Fu R. Zhang Y. H. Liu Z. Chen Z. H. Xu J. (2022). Single-cell RNA sequencing unravels upregulation of immune cell crosstalk in relapsed pediatric ependymoma. Front. Immunol. 13, 903246. 10.3389/fimmu.2022.903246 35844565 PMC9281506

[B177] Wu Y. Fletcher M. Gu Z. Wang Q. Costa B. Bertoni A. (2020). Glioblastoma epigenome profiling identifies SOX10 as a master regulator of molecular tumour subtype. Nat. Commun. 11, 6434. 10.1038/s41467-020-20225-w 33339831 PMC7749178

[B178] Xia C. Babcock H. P. Moffitt J. R. Zhuang X. (2019). Multiplexed detection of RNA using MERFISH and branched DNA amplification. Sci. Rep. 9, 7721. 10.1038/s41598-019-43943-8 31118500 PMC6531529

[B179] Xu W. Wen Y. Liang Y. Xu Q. Wang X. Jin W. (2021). A plate-based single-cell ATAC-seq workflow for fast and robust profiling of chromatin accessibility. Nat. Protoc. 16, 4084–4107. 10.1038/s41596-021-00583-5 34282334

[B180] Yuan J. Levitin H. M. Frattini V. Bush E. C. Boyett D. M. Samanamud J. (2018). Single-cell transcriptome analysis of lineage diversity in high-grade glioma. Genome Med. 10, 57. 10.1186/s13073-018-0567-9 30041684 PMC6058390

[B181] Zhai Y. Li G. Li R. Chang Y. Feng Y. Wang D. (2020). Single-cell RNA-sequencing shift in the interaction pattern between glioma stem cells and immune cells during tumorigenesis. Front. Immunol. 11, 581209. 10.3389/fimmu.2020.581209 33133100 PMC7580180

[B182] Zhang Q. Cheng S. Wang Y. Wang M. Lu Y. Wen Z. (2021). Interrogation of the microenvironmental landscape in spinal ependymomas reveals dual functions of tumor-associated macrophages. Nat. Commun. 12, 6867. 10.1038/s41467-021-27018-9 34824203 PMC8617028

[B183] Zhou H. M. Zhang J. G. Zhang X. Li Q. (2021). Targeting cancer stem cells for reversing therapy resistance: Mechanism, signaling, and prospective agents. Signal Transduct. Target Ther. 6, 62. 10.1038/s41392-020-00430-1 33589595 PMC7884707

